# Search for top squark pair production using dilepton final states in ${\text {p}}{\text {p}}$ collision data collected at $\sqrt{s}=13\,\text {TeV}$

**DOI:** 10.1140/epjc/s10052-020-08701-5

**Published:** 2021-01-05

**Authors:** A. M. Sirunyan, A. Tumasyan, W. Adam, F. Ambrogi, T. Bergauer, M. Dragicevic, J. Erö, A. Escalante Del Valle, R. Frühwirth, M. Jeitler, N. Krammer, L. Lechner, D. Liko, T. Madlener, I. Mikulec, F. M. Pitters, N. Rad, J. Schieck, R. Schöfbeck, M. Spanring, S. Templ, W. Waltenberger, C.-E. Wulz, M. Zarucki, V. Chekhovsky, A. Litomin, V. Makarenko, J. Suarez Gonzalez, M. R. Darwish, E. A. De Wolf, D. Di Croce, X. Janssen, T. Kello, A. Lelek, M. Pieters, H. Rejeb Sfar, H. Van Haevermaet, P. Van Mechelen, S. Van Putte, N. Van Remortel, F. Blekman, E. S. Bols, S. S. Chhibra, J. D’Hondt, J. De Clercq, D. Lontkovskyi, S. Lowette, I. Marchesini, S. Moortgat, A. Morton, Q. Python, S. Tavernier, W. Van Doninck, P. Van Mulders, D. Beghin, B. Bilin, B. Clerbaux, G. De Lentdecker, B. Dorney, L. Favart, A. Grebenyuk, A. K. Kalsi, I. Makarenko, L. Moureaux, L. Pétré, A. Popov, N. Postiau, E. Starling, L. Thomas, C. Vander Velde, P. Vanlaer, D. Vannerom, L. Wezenbeek, T. Cornelis, D. Dobur, M. Gruchala, I. Khvastunov, M. Niedziela, C. Roskas, K. Skovpen, M. Tytgat, W. Verbeke, B. Vermassen, M. Vit, G. Bruno, F. Bury, C. Caputo, P. David, C. Delaere, M. Delcourt, I. S. Donertas, A. Giammanco, V. Lemaitre, K. Mondal, J. Prisciandaro, A. Taliercio, M. Teklishyn, P. Vischia, S. Wuyckens, J. Zobec, G. A. Alves, G. Correia Silva, C. Hensel, A. Moraes, W. L. Aldá Júnior, E. Belchior Batista Das Chagas, H. BRANDAO MALBOUISSON, W. Carvalho, J. Chinellato, E. Coelho, E. M. Da Costa, G. G. Da Silveira, D. De Jesus Damiao, S. Fonseca De Souza, J. Martins, D. Matos Figueiredo, M. Medina Jaime, M. Melo De Almeida, C. Mora Herrera, L. Mundim, H. Nogima, P. Rebello Teles, L. J. Sanchez Rosas, A. Santoro, S. M. Silva Do Amaral, A. Sznajder, M. Thiel, E. J. Tonelli Manganote, F. Torres Da Silva De Araujo, A. Vilela Pereira, C. A. Bernardes, L. Calligaris, T. R. Fernandez Perez Tomei, E. M. Gregores, D. S. Lemos, P. G. Mercadante, S. F. Novaes, Sandra S. Padula, A. Aleksandrov, G. Antchev, I. Atanasov, R. Hadjiiska, P. Iaydjiev, M. Misheva, M. Rodozov, M. Shopova, G. Sultanov, M. Bonchev, A. Dimitrov, T. Ivanov, L. Litov, B. Pavlov, P. Petkov, A. Petrov, W. Fang, Q. Guo, H. Wang, L. Yuan, M. Ahmad, Z. Hu, Y. Wang, E. Chapon, G. M. Chen, H. S. Chen, M. Chen, A. Kapoor, D. Leggat, H. Liao, Z. Liu, R. Sharma, A. Spiezia, J. Tao, J. Thomas-wilsker, J. Wang, H. Zhang, S. Zhang, J. Zhao, A. Agapitos, Y. Ban, C. Chen, Q. Huang, A. Levin, Q. Li, M. Lu, X. Lyu, Y. Mao, S. J. Qian, D. Wang, Q. Wang, J. Xiao, Z. You, X. Gao, M. Xiao, C. Avila, A. Cabrera, C. Florez, J. Fraga, A. Sarkar, M. A. Segura Delgado, J. Jaramillo, J. Mejia Guisao, F. Ramirez, J. D. Ruiz Alvarez, C. A. Salazar González, N. Vanegas Arbelaez, D. Giljanovic, N. Godinovic, D. Lelas, I. Puljak, T. Sculac, Z. Antunovic, M. Kovac, V. Brigljevic, D. Ferencek, D. Majumder, M. Roguljic, A. Starodumov, T. Susa, M. W. Ather, A. Attikis, E. Erodotou, A. Ioannou, G. Kole, M. Kolosova, S. Konstantinou, G. Mavromanolakis, J. Mousa, C. Nicolaou, F. Ptochos, P. A. Razis, H. Rykaczewski, H. Saka, D. Tsiakkouri, M. Finger, M. Finger, A. Kveton, J. Tomsa, E. Ayala, E. Carrera Jarrin, H. Abdalla, Y. Assran, S. Khalil, M. A. Mahmoud, Y. Mohammed, S. Bhowmik, A. Carvalho Antunes De Oliveira, R. K. Dewanjee, K. Ehataht, M. Kadastik, M. Raidal, C. Veelken, P. Eerola, L. Forthomme, H. Kirschenmann, K. Osterberg, M. Voutilainen, E. Brücken, F. Garcia, J. Havukainen, V. Karimäki, M. S. Kim, R. Kinnunen, T. Lampén, K. Lassila-Perini, S. Laurila, S. Lehti, T. Lindén, H. Siikonen, E. Tuominen, J. Tuominiemi, P. Luukka, T. Tuuva, C. Amendola, M. Besancon, F. Couderc, M. Dejardin, D. Denegri, J. L. Faure, F. Ferri, S. Ganjour, A. Givernaud, P. Gras, G. Hamel de Monchenault, P. Jarry, B. Lenzi, E. Locci, J. Malcles, J. Rander, A. Rosowsky, M.Ö. Sahin, A. Savoy-Navarro, M. Titov, G. B. Yu, S. Ahuja, F. Beaudette, M. Bonanomi, A. Buchot Perraguin, P. Busson, C. Charlot, O. Davignon, B. Diab, G. Falmagne, R. Granier de Cassagnac, A. Hakimi, I. Kucher, A. Lobanov, C. Martin Perez, M. Nguyen, C. Ochando, P. Paganini, J. Rembser, R. Salerno, J. B. Sauvan, Y. Sirois, A. Zabi, A. Zghiche, J.-L. Agram, J. Andrea, D. Bloch, G. Bourgatte, J.-M. Brom, E. C. Chabert, C. Collard, J.-C. Fontaine, D. Gelé, U. Goerlach, C. Grimault, A.-C. Le Bihan, P. Van Hove, E. Asilar, S. Beauceron, C. Bernet, G. Boudoul, C. Camen, A. Carle, N. Chanon, D. Contardo, P. Depasse, H. El Mamouni, J. Fay, S. Gascon, M. Gouzevitch, B. Ille, Sa. Jain, I. B. Laktineh, H. Lattaud, A. Lesauvage, M. Lethuillier, L. Mirabito, L. Torterotot, G. Touquet, M. Vander Donckt, S. Viret, I. Bagaturia, Z. Tsamalaidze, L. Feld, K. Klein, M. Lipinski, D. Meuser, A. Pauls, M. Preuten, M. P. Rauch, J. Schulz, M. Teroerde, D. Eliseev, M. Erdmann, P. Fackeldey, B. Fischer, S. Ghosh, T. Hebbeker, K. Hoepfner, H. Keller, L. Mastrolorenzo, M. Merschmeyer, A. Meyer, P. Millet, G. Mocellin, S. Mondal, S. Mukherjee, D. Noll, A. Novak, T. Pook, A. Pozdnyakov, T. Quast, M. Radziej, Y. Rath, H. Reithler, J. Roemer, A. Schmidt, S. C. Schuler, A. Sharma, S. Wiedenbeck, S. Zaleski, C. Dziwok, G. Flügge, W. Haj Ahmad, O. Hlushchenko, T. Kress, A. Nowack, C. Pistone, O. Pooth, D. Roy, H. Sert, A. Stahl, T. Ziemons, H. Aarup Petersen, M. Aldaya Martin, P. Asmuss, I. Babounikau, S. Baxter, O. Behnke, A. Bermúdez Martínez, A. A. Bin Anuar, K. Borras, V. Botta, D. Brunner, A. Campbell, A. Cardini, P. Connor, S. Consuegra Rodríguez, V. Danilov, A. De Wit, M. M. Defranchis, L. Didukh, D. Domínguez Damiani, G. Eckerlin, D. Eckstein, T. Eichhorn, L. I. Estevez Banos, E. Gallo, A. Geiser, A. Giraldi, A. Grohsjean, M. Guthoff, A. Harb, A. Jafari, N. Z. Jomhari, H. Jung, A. Kasem, M. Kasemann, H. Kaveh, C. Kleinwort, J. Knolle, D. Krücker, W. Lange, T. Lenz, J. Lidrych, K. Lipka, W. Lohmann, R. Mankel, I.-A. Melzer-Pellmann, J. Metwally, A. B. Meyer, M. Meyer, M. Missiroli, J. Mnich, A. Mussgiller, V. Myronenko, Y. Otarid, D. Pérez Adán, S. K. Pflitsch, D. Pitzl, A. Raspereza, A. Saggio, A. Saibel, M. Savitskyi, V. Scheurer, P. Schütze, C. Schwanenberger, A. Singh, R. E. Sosa Ricardo, N. Tonon, O. Turkot, A. Vagnerini, M. Van De Klundert, R. Walsh, D. Walter, Y. Wen, K. Wichmann, C. Wissing, S. Wuchterl, O. Zenaiev, R. Zlebcik, R. Aggleton, S. Bein, L. Benato, A. Benecke, K. De Leo, T. Dreyer, A. Ebrahimi, M. Eich, F. Feindt, A. Fröhlich, C. Garbers, E. Garutti, P. Gunnellini, J. Haller, A. Hinzmann, A. Karavdina, G. Kasieczka, R. Klanner, R. Kogler, V. Kutzner, J. Lange, T. Lange, A. Malara, C. E. N. Niemeyer, A. Nigamova, K. J. Pena Rodriguez, O. Rieger, P. Schleper, S. Schumann, J. Schwandt, D. Schwarz, J. Sonneveld, H. Stadie, G. Steinbrück, B. Vormwald, I. Zoi, M. Baselga, S. Baur, J. Bechtel, T. Berger, E. Butz, R. Caspart, T. Chwalek, W. De Boer, A. Dierlamm, A. Droll, K. El Morabit, N. Faltermann, K. Flöh, M. Giffels, A. Gottmann, F. Hartmann, C. Heidecker, U. Husemann, M. A. Iqbal, I. Katkov, P. Keicher, R. Koppenhöfer, S. Maier, M. Metzler, S. Mitra, D. Müller, Th. Müller, M. Musich, G. Quast, K. Rabbertz, J. Rauser, D. Savoiu, D. Schäfer, M. Schnepf, M. Schröder, D. Seith, I. Shvetsov, H. J. Simonis, R. Ulrich, M. Wassmer, M. Weber, R. Wolf, S. Wozniewski, G. Anagnostou, P. Asenov, G. Daskalakis, T. Geralis, A. Kyriakis, D. Loukas, G. Paspalaki, A. Stakia, M. Diamantopoulou, D. Karasavvas, G. Karathanasis, P. Kontaxakis, C. K. Koraka, A. Manousakis-katsikakis, A. Panagiotou, I. Papavergou, N. Saoulidou, K. Theofilatos, K. Vellidis, E. Vourliotis, G. Bakas, K. Kousouris, I. Papakrivopoulos, G. Tsipolitis, A. Zacharopoulou, I. Evangelou, C. Foudas, P. Gianneios, P. Katsoulis, P. Kokkas, S. Mallios, K. Manitara, N. Manthos, I. Papadopoulos, J. Strologas, M. Bartók, R. Chudasama, M. Csanad, M. M. A. Gadallah, S. Lökös, P. Major, K. Mandal, A. Mehta, G. Pasztor, O. Surányi, G. I. Veres, G. Bencze, C. Hajdu, D. Horvath, F. Sikler, V. Veszpremi, G. Vesztergombi, S. Czellar, J. Karancsi, J. Molnar, Z. Szillasi, D. Teyssier, P. Raics, Z. L. Trocsanyi, G. Zilizi, T. Csorgo, F. Nemes, T. Novak, S. Choudhury, J. R. Komaragiri, D. Kumar, L. Panwar, P. C. Tiwari, S. Bahinipati, D. Dash, C. Kar, P. Mal, T. Mishra, V. K. Muraleedharan Nair Bindhu, A. Nayak, D. K. Sahoo, N. Sur, S. K. Swain, S. Bansal, S. B. Beri, V. Bhatnagar, S. Chauhan, N. Dhingra, R. Gupta, A. Kaur, S. Kaur, P. Kumari, M. Lohan, M. Meena, K. Sandeep, S. Sharma, J. B. Singh, A. K. Virdi, A. Ahmed, A. Bhardwaj, B. C. Choudhary, R. B. Garg, M. Gola, S. Keshri, A. Kumar, M. Naimuddin, P. Priyanka, K. Ranjan, A. Shah, M. Bharti, R. Bhattacharya, S. Bhattacharya, D. Bhowmik, S. Dutta, S. Ghosh, B. Gomber, M. Maity, S. Nandan, P. Palit, A. Purohit, P. K. Rout, G. Saha, S. Sarkar, M. Sharan, B. Singh, S. Thakur, P. K. Behera, S. C. Behera, P. Kalbhor, A. Muhammad, R. Pradhan, P. R. Pujahari, A. Sharma, A. K. Sikdar, D. Dutta, V. Kumar, K. Naskar, P. K. Netrakanti, L. M. Pant, P. Shukla, T. Aziz, M. A. Bhat, S. Dugad, R. Kumar Verma, G. B. Mohanty, U. Sarkar, S. Banerjee, S. Bhattacharya, S. Chatterjee, M. Guchait, S. Karmakar, S. Kumar, G. Majumder, K. Mazumdar, S. Mukherjee, D. Roy, N. Sahoo, S. Dube, B. Kansal, K. Kothekar, S. Pandey, A. Rane, A. Rastogi, S. Sharma, H. Bakhshiansohi, S. Chenarani, S. M. Etesami, M. Khakzad, M. Mohammadi Najafabadi, M. Felcini, M. Grunewald, M. Abbrescia, R. Aly, C. Aruta, A. Colaleo, D. Creanza, N. De Filippis, M. De Palma, A. Di Florio, A. Di Pilato, W. Elmetenawee, L. Fiore, A. Gelmi, M. Gul, G. Iaselli, M. Ince, S. Lezki, G. Maggi, M. Maggi, I. Margjeka, V. Mastrapasqua, J. A. Merlin, S. My, S. Nuzzo, A. Pompili, G. Pugliese, A. Ranieri, G. Selvaggi, L. Silvestris, F. M. Simone, R. Venditti, P. Verwilligen, G. Abbiendi, C. Battilana, D. Bonacorsi, L. Borgonovi, S. Braibant-Giacomelli, R. Campanini, P. Capiluppi, A. Castro, F. R. Cavallo, M. Cuffiani, G. M. Dallavalle, T. Diotalevi, F. Fabbri, A. Fanfani, E. Fontanesi, P. Giacomelli, L. Giommi, C. Grandi, L. Guiducci, F. Iemmi, S. Lo Meo, S. Marcellini, G. Masetti, F. L. Navarria, A. Perrotta, F. Primavera, A. M. Rossi, T. Rovelli, G. P. Siroli, N. Tosi, S. Albergo, S. Costa, A. Di Mattia, R. Potenza, A. Tricomi, C. Tuve, G. Barbagli, A. Cassese, R. Ceccarelli, V. Ciulli, C. Civinini, R. D’Alessandro, F. Fiori, E. Focardi, G. Latino, P. Lenzi, M. Lizzo, M. Meschini, S. Paoletti, R. Seidita, G. Sguazzoni, L. Viliani, L. Benussi, S. Bianco, D. Piccolo, M. Bozzo, F. Ferro, R. Mulargia, E. Robutti, S. Tosi, A. Benaglia, A. Beschi, F. Brivio, F. Cetorelli, V. Ciriolo, F. De Guio, M. E. Dinardo, P. Dini, S. Gennai, A. Ghezzi, P. Govoni, L. Guzzi, M. Malberti, S. Malvezzi, D. Menasce, F. Monti, L. Moroni, M. Paganoni, D. Pedrini, S. Ragazzi, T. Tabarelli de Fatis, D. Valsecchi, D. Zuolo, S. Buontempo, N. Cavallo, A. De Iorio, F. Fabozzi, F. Fienga, A. O. M. Iorio, L. Lista, S. Meola, P. Paolucci, B. Rossi, C. Sciacca, E. Voevodina, P. Azzi, N. Bacchetta, D. Bisello, A. Boletti, A. Bragagnolo, R. Carlin, P. Checchia, P. De Castro Manzano, T. Dorigo, F. Gasparini, U. Gasparini, S. Y. Hoh, L. Layer, M. Margoni, A. T. Meneguzzo, M. Presilla, P. Ronchese, R. Rossin, F. Simonetto, G. Strong, A. Tiko, M. Tosi, H. YARAR, M. Zanetti, P. Zotto, A. Zucchetta, G. Zumerle, C. Aime‘, A. Braghieri, S. Calzaferri, D. Fiorina, P. Montagna, S. P. Ratti, V. Re, M. Ressegotti, C. Riccardi, P. Salvini, I. Vai, P. Vitulo, M. Biasini, G. M. Bilei, D. Ciangottini, L. Fanò, P. Lariccia, G. Mantovani, V. Mariani, M. Menichelli, F. Moscatelli, A. Piccinelli, A. Rossi, A. Santocchia, D. Spiga, T. Tedeschi, K. Androsov, P. Azzurri, G. Bagliesi, V. Bertacchi, L. Bianchini, T. Boccali, R. Castaldi, M. A. Ciocci, R. Dell’Orso, M. R. Di Domenico, S. Donato, L. Giannini, A. Giassi, M. T. Grippo, F. Ligabue, E. Manca, G. Mandorli, A. Messineo, F. Palla, G. Ramirez-Sanchez, A. Rizzi, G. Rolandi, S. Roy Chowdhury, A. Scribano, N. Shafiei, P. Spagnolo, R. Tenchini, G. Tonelli, N. Turini, A. Venturi, P. G. Verdini, F. Cavallari, M. Cipriani, D. Del Re, E. Di Marco, M. Diemoz, E. Longo, P. Meridiani, G. Organtini, F. Pandolfi, R. Paramatti, C. Quaranta, S. Rahatlou, C. Rovelli, F. Santanastasio, L. Soffi, R. Tramontano, N. Amapane, R. Arcidiacono, S. Argiro, M. Arneodo, N. Bartosik, R. Bellan, A. Bellora, C. Biino, A. Cappati, N. Cartiglia, S. Cometti, M. Costa, R. Covarelli, N. Demaria, B. Kiani, F. Legger, C. Mariotti, S. Maselli, E. Migliore, V. Monaco, E. Monteil, M. Monteno, M. M. Obertino, G. Ortona, L. Pacher, N. Pastrone, M. Pelliccioni, G. L. Pinna Angioni, M. Ruspa, R. Salvatico, F. Siviero, V. Sola, A. Solano, D. Soldi, A. Staiano, D. Trocino, S. Belforte, V. Candelise, M. Casarsa, F. Cossutti, A. Da Rold, G. Della Ricca, F. Vazzoler, S. Dogra, C. Huh, B. Kim, D. H. Kim, G. N. Kim, J. Lee, S. W. Lee, C. S. Moon, Y. D. Oh, S. I. Pak, B. C. Radburn-Smith, S. Sekmen, Y. C. Yang, H. Kim, D. H. Moon, B. Francois, T. J. Kim, J. Park, S. Cho, S. Choi, Y. Go, S. Ha, B. Hong, K. Lee, K. S. Lee, J. Lim, J. Park, S. K. Park, J. Yoo, J. Goh, A. Gurtu, H. S. Kim, Y. Kim, J. Almond, J. H. Bhyun, J. Choi, S. Jeon, J. Kim, J. S. Kim, S. Ko, H. Kwon, H. Lee, K. Lee, S. Lee, K. Nam, B. H. Oh, M. Oh, S. B. Oh, H. Seo, U. K. Yang, I. Yoon, D. Jeon, J. H. Kim, B. Ko, J. S. H. Lee, I. C. Park, Y. Roh, D. Song, I. J. Watson, H. D. Yoo, Y. Choi, C. Hwang, Y. Jeong, H. Lee, Y. Lee, I. Yu, V. Veckalns, A. Juodagalvis, A. Rinkevicius, G. Tamulaitis, W. A. T. Wan Abdullah, M. N. Yusli, Z. Zolkapli, J. F. Benitez, A. Castaneda Hernandez, J. A. Murillo Quijada, L. Valencia Palomo, G. Ayala, H. Castilla-Valdez, E. De La Cruz-Burelo, I. Heredia-De La Cruz, R. Lopez-Fernandez, D. A. Perez Navarro, A. Sanchez-Hernandez, S. Carrillo Moreno, C. Oropeza Barrera, M. Ramirez-Garcia, F. Vazquez Valencia, J. Eysermans, I. Pedraza, H. A. Salazar Ibarguen, C. Uribe Estrada, A. Morelos Pineda, J. Mijuskovic, N. Raicevic, D. Krofcheck, S. Bheesette, P. H. Butler, A. Ahmad, M. I. Asghar, M. I. M. Awan, H. R. Hoorani, W. A. Khan, M. A. Shah, M. Shoaib, M. Waqas, V. Avati, L. Grzanka, M. Malawski, H. Bialkowska, M. Bluj, B. Boimska, T. Frueboes, M. Górski, M. Kazana, M. Szleper, P. Traczyk, P. Zalewski, K. Bunkowski, A. Byszuk, K. Doroba, A. Kalinowski, M. Konecki, J. Krolikowski, M. Olszewski, M. Walczak, M. Araujo, P. Bargassa, D. Bastos, P. Faccioli, M. Gallinaro, J. Hollar, N. Leonardo, T. Niknejad, J. Seixas, K. Shchelina, O. Toldaiev, J. Varela, S. Afanasiev, P. Bunin, M. Gavrilenko, I. Golutvin, I. Gorbunov, A. Kamenev, V. Karjavine, A. Lanev, A. Malakhov, V. Matveev, P. Moisenz, V. Palichik, V. Perelygin, M. Savina, D. Seitova, V. Shalaev, S. Shmatov, S. Shulha, V. Smirnov, O. Teryaev, N. Voytishin, A. Zarubin, I. Zhizhin, G. Gavrilov, V. Golovtcov, Y. Ivanov, V. Kim, E. Kuznetsova, V. Murzin, V. Oreshkin, I. Smirnov, D. Sosnov, V. Sulimov, L. Uvarov, S. Volkov, A. Vorobyev, Yu. Andreev, A. Dermenev, S. Gninenko, N. Golubev, A. Karneyeu, M. Kirsanov, N. Krasnikov, A. Pashenkov, G. Pivovarov, D. Tlisov, A. Toropin, V. Epshteyn, V. Gavrilov, N. Lychkovskaya, A. Nikitenko, V. Popov, G. Safronov, A. Spiridonov, A. Stepennov, M. Toms, E. Vlasov, A. Zhokin, T. Aushev, O. Bychkova, M. Danilov, D. Philippov, E. Popova, V. Rusinov, V. Andreev, M. Azarkin, I. Dremin, M. Kirakosyan, A. Terkulov, A. Belyaev, E. Boos, V. Bunichev, M. Dubinin, L. Dudko, A. Ershov, A. Gribushin, V. Klyukhin, O. Kodolova, I. Lokhtin, S. Obraztsov, M. Perfilov, V. Savrin, V. Blinov, T. Dimova, L. Kardapoltsev, I. Ovtin, Y. Skovpen, I. Azhgirey, I. Bayshev, V. Kachanov, A. Kalinin, D. Konstantinov, V. Petrov, R. Ryutin, A. Sobol, S. Troshin, N. Tyurin, A. Uzunian, A. Volkov, A. Babaev, A. Iuzhakov, V. Okhotnikov, L. Sukhikh, V. Borchsh, V. Ivanchenko, E. Tcherniaev, P. Adzic, P. Cirkovic, M. Dordevic, P. Milenovic, J. Milosevic, M. Aguilar-Benitez, J. Alcaraz Maestre, A. Álvarez Fernández, I. Bachiller, M. Barrio Luna, Cristina F. Bedoya, J. A. Brochero Cifuentes, C. A. Carrillo Montoya, M. Cepeda, M. Cerrada, N. Colino, B. De La Cruz, A. Delgado Peris, J. P. Fernández Ramos, J. Flix, M. C. Fouz, A. García Alonso, O. Gonzalez Lopez, S. Goy Lopez, J. M. Hernandez, M. I. Josa, J. León Holgado, D. Moran, Á. Navarro Tobar, A. Pérez-Calero Yzquierdo, J. Puerta Pelayo, I. Redondo, L. Romero, S. Sánchez Navas, M. S. Soares, A. Triossi, L. Urda Gómez, C. Willmott, C. Albajar, J. F. de Trocóniz, R. Reyes-Almanza, B. Alvarez Gonzalez, J. Cuevas, C. Erice, J. Fernandez Menendez, S. Folgueras, I. Gonzalez Caballero, E. Palencia Cortezon, C. Ramón Álvarez, J. Ripoll Sau, V. Rodríguez Bouza, S. Sanchez Cruz, A. Trapote, I. J. Cabrillo, A. Calderon, B. Chazin Quero, J. Duarte Campderros, M. Fernandez, P. J. Fernández Manteca, G. Gomez, C. Martinez Rivero, P. Martinez Ruiz del Arbol, F. Matorras, J. Piedra Gomez, C. Prieels, F. Ricci-Tam, T. Rodrigo, A. Ruiz-Jimeno, L. Scodellaro, I. Vila, J. M. Vizan Garcia, M. K. Jayananda, B. Kailasapathy, D. U. J. Sonnadara, D. D. C. Wickramarathna, W. G. D. Dharmaratna, K. Liyanage, N. Perera, N. Wickramage, T. K. Aarrestad, D. Abbaneo, B. Akgun, E. Auffray, G. Auzinger, J. Baechler, P. Baillon, A. H. Ball, D. Barney, J. Bendavid, N. Beni, M. Bianco, A. Bocci, P. Bortignon, E. Bossini, E. Brondolin, T. Camporesi, G. Cerminara, L. Cristella, D. d’Enterria, A. Dabrowski, N. Daci, V. Daponte, A. David, A. De Roeck, M. Deile, R. Di Maria, M. Dobson, M. Dünser, N. Dupont, A. Elliott-Peisert, N. Emriskova, F. Fallavollita, D. Fasanella, S. Fiorendi, A. Florent, G. Franzoni, J. Fulcher, W. Funk, S. Giani, D. Gigi, K. Gill, F. Glege, L. Gouskos, M. Guilbaud, D. Gulhan, M. Haranko, J. Hegeman, Y. Iiyama, V. Innocente, T. James, P. Janot, J. Kaspar, J. Kieseler, M. Komm, N. Kratochwil, C. Lange, P. Lecoq, K. Long, C. Lourenço, L. Malgeri, M. Mannelli, A. Massironi, F. Meijers, S. Mersi, E. Meschi, F. Moortgat, M. Mulders, J. Ngadiuba, J. Niedziela, S. Orfanelli, L. Orsini, F. Pantaleo, L. Pape, E. Perez, M. Peruzzi, A. Petrilli, G. Petrucciani, A. Pfeiffer, M. Pierini, D. Rabady, A. Racz, M. Rieger, M. Rovere, H. Sakulin, J. Salfeld-Nebgen, S. Scarfi, C. Schäfer, C. Schwick, M. Selvaggi, A. Sharma, P. Silva, W. Snoeys, P. Sphicas, J. Steggemann, S. Summers, V. R. Tavolaro, D. Treille, A. Tsirou, G. P. Van Onsem, A. Vartak, M. Verzetti, K. A. Wozniak, W. D. Zeuner, L. Caminada, W. Erdmann, R. Horisberger, Q. Ingram, H. C. Kaestli, D. Kotlinski, U. Langenegger, T. Rohe, M. Backhaus, P. Berger, A. Calandri, N. Chernyavskaya, A. De Cosa, G. Dissertori, M. Dittmar, M. Donegà, C. Dorfer, T. Gadek, T. A. Gómez Espinosa, C. Grab, D. Hits, W. Lustermann, A.-M. Lyon, R. A. Manzoni, M. T. Meinhard, F. Micheli, F. Nessi-Tedaldi, F. Pauss, V. Perovic, G. Perrin, L. Perrozzi, S. Pigazzini, M. G. Ratti, M. Reichmann, C. Reissel, T. Reitenspiess, B. Ristic, D. Ruini, D. A. Sanz Becerra, M. Schönenberger, V. Stampf, M. L. Vesterbacka Olsson, R. Wallny, D. H. Zhu, C. Amsler, C. Botta, D. Brzhechko, M. F. Canelli, R. Del Burgo, J. K. Heikkilä, M. Huwiler, A. Jofrehei, B. Kilminster, S. Leontsinis, A. Macchiolo, P. Meiring, V. M. Mikuni, U. Molinatti, I. Neutelings, G. Rauco, A. Reimers, P. Robmann, K. Schweiger, Y. Takahashi, S. Wertz, C. Adloff, C. M. Kuo, W. Lin, A. Roy, T. Sarkar, S. S. Yu, L. Ceard, P. Chang, Y. Chao, K. F. Chen, P. H. Chen, W.-S. Hou, Y. y. Li, R.-S. Lu, E. Paganis, A. Psallidas, A. Steen, E. Yazgan, B. Asavapibhop, C. Asawatangtrakuldee, N. Srimanobhas, F. Boran, S. Damarseckin, Z. S. Demiroglu, F. Dolek, C. Dozen, I. Dumanoglu, E. Eskut, G. Gokbulut, Y. Guler, E. Gurpinar Guler, I. Hos, C. Isik, E. E. Kangal, O. Kara, A. Kayis Topaksu, U. Kiminsu, G. Onengut, K. Ozdemir, A. Polatoz, A. E. Simsek, B. Tali, U. G. Tok, S. Turkcapar, I. S. Zorbakir, C. Zorbilmez, B. Isildak, G. Karapinar, K. Ocalan, M. Yalvac, I. O. Atakisi, E. Gülmez, M. Kaya, O. Kaya, Ö. Özçelik, S. Tekten, E. A. Yetkin, A. Cakir, K. Cankocak, Y. Komurcu, S. Sen, F. Aydogmus Sen, S. Cerci, B. Kaynak, S. Ozkorucuklu, D. Sunar Cerci, B. Grynyov, L. Levchuk, E. Bhal, S. Bologna, J. J. Brooke, E. Clement, D. Cussans, H. Flacher, J. Goldstein, G. P. Heath, H. F. Heath, L. Kreczko, B. Krikler, S. Paramesvaran, T. Sakuma, S. Seif El Nasr-Storey, V. J. Smith, J. Taylor, A. Titterton, K. W. Bell, A. Belyaev, C. Brew, R. M. Brown, D. J. A. Cockerill, K. V. Ellis, K. Harder, S. Harper, J. Linacre, K. Manolopoulos, D. M. Newbold, E. Olaiya, D. Petyt, T. Reis, T. Schuh, C. H. Shepherd-Themistocleous, A. Thea, I. R. Tomalin, T. Williams, R. Bainbridge, P. Bloch, S. Bonomally, J. Borg, S. Breeze, O. Buchmuller, A. Bundock, V. Cepaitis, G. S. Chahal, D. Colling, P. Dauncey, G. Davies, M. Della Negra, G. Fedi, G. Hall, G. Iles, J. Langford, L. Lyons, A.-M. Magnan, S. Malik, A. Martelli, V. Milosevic, J. Nash, V. Palladino, M. Pesaresi, D. M. Raymond, A. Richards, A. Rose, E. Scott, C. Seez, A. Shtipliyski, M. Stoye, A. Tapper, K. Uchida, T. Virdee, N. Wardle, S. N. Webb, D. Winterbottom, A. G. Zecchinelli, J. E. Cole, P. R. Hobson, A. Khan, P. Kyberd, C. K. Mackay, I. D. Reid, L. Teodorescu, S. Zahid, A. Brinkerhoff, K. Call, B. Caraway, J. Dittmann, K. Hatakeyama, A. R. Kanuganti, C. Madrid, B. McMaster, N. Pastika, S. Sawant, C. Smith, J. Wilson, R. Bartek, A. Dominguez, R. Uniyal, A. M. Vargas Hernandez, A. Buccilli, O. Charaf, S. I. Cooper, S. V. Gleyzer, C. Henderson, P. Rumerio, C. West, A. Akpinar, A. Albert, D. Arcaro, C. Cosby, Z. Demiragli, D. Gastler, C. Richardson, J. Rohlf, K. Salyer, D. Sperka, D. Spitzbart, I. Suarez, S. Yuan, D. Zou, G. Benelli, B. Burkle, X. Coubez, D. Cutts, Y. t. Duh, M. Hadley, U. Heintz, J. M. Hogan, K. H. M. Kwok, E. Laird, G. Landsberg, K. T. Lau, J. Lee, M. Narain, S. Sagir, R. Syarif, E. Usai, W. Y. Wong, D. Yu, W. Zhang, R. Band, C. Brainerd, R. Breedon, M. Calderon De La Barca Sanchez, M. Chertok, J. Conway, R. Conway, P. T. Cox, R. Erbacher, C. Flores, G. Funk, F. Jensen, W. Ko, O. Kukral, R. Lander, M. Mulhearn, D. Pellett, J. Pilot, M. Shi, D. Taylor, K. Tos, M. Tripathi, Y. Yao, F. Zhang, M. Bachtis, R. Cousins, A. Dasgupta, D. Hamilton, J. Hauser, M. Ignatenko, T. Lam, N. Mccoll, W. A. Nash, S. Regnard, D. Saltzberg, C. Schnaible, B. Stone, V. Valuev, K. Burt, Y. Chen, R. Clare, J. W. Gary, S. M. A. Ghiasi Shirazi, G. Hanson, G. Karapostoli, O. R. Long, N. Manganelli, M. Olmedo Negrete, M. I. Paneva, W. Si, S. Wimpenny, Y. Zhang, J. G. Branson, P. Chang, S. Cittolin, S. Cooperstein, N. Deelen, M. Derdzinski, J. Duarte, R. Gerosa, D. Gilbert, B. Hashemi, V. Krutelyov, J. Letts, M. Masciovecchio, S. May, S. Padhi, M. Pieri, V. Sharma, M. Tadel, F. Würthwein, A. Yagil, N. Amin, C. Campagnari, M. Citron, A. Dorsett, V. Dutta, J. Incandela, B. Marsh, H. Mei, A. Ovcharova, H. Qu, M. Quinnan, J. Richman, U. Sarica, D. Stuart, S. Wang, D. Anderson, A. Bornheim, O. Cerri, I. Dutta, J. M. Lawhorn, N. Lu, J. Mao, H. B. Newman, T. Q. Nguyen, J. Pata, M. Spiropulu, J. R. Vlimant, S. Xie, Z. Zhang, R. Y. Zhu, J. Alison, M. B. Andrews, T. Ferguson, T. Mudholkar, M. Paulini, M. Sun, I. Vorobiev, J. P. Cumalat, W. T. Ford, E. MacDonald, T. Mulholland, R. Patel, A. Perloff, K. Stenson, K. A. Ulmer, S. R. Wagner, J. Alexander, Y. Cheng, J. Chu, D. J. Cranshaw, A. Datta, A. Frankenthal, K. Mcdermott, J. Monroy, J. R. Patterson, D. Quach, A. Ryd, W. Sun, S. M. Tan, Z. Tao, J. Thom, P. Wittich, M. Zientek, S. Abdullin, M. Albrow, M. Alyari, G. Apollinari, A. Apresyan, A. Apyan, S. Banerjee, L. A. T. Bauerdick, A. Beretvas, D. Berry, J. Berryhill, P. C. Bhat, K. Burkett, J. N. Butler, A. Canepa, G. B. Cerati, H. W. K. Cheung, F. Chlebana, M. Cremonesi, V. D. Elvira, J. Freeman, Z. Gecse, E. Gottschalk, L. Gray, D. Green, S. Grünendahl, O. Gutsche, R. M. Harris, S. Hasegawa, R. Heller, T. C. Herwig, J. Hirschauer, B. Jayatilaka, S. Jindariani, M. Johnson, U. Joshi, P. Klabbers, T. Klijnsma, B. Klima, M. J. Kortelainen, S. Lammel, D. Lincoln, R. Lipton, M. Liu, T. Liu, J. Lykken, K. Maeshima, D. Mason, P. McBride, P. Merkel, S. Mrenna, S. Nahn, V. O’Dell, V. Papadimitriou, K. Pedro, C. Pena, O. Prokofyev, F. Ravera, A. Reinsvold Hall, L. Ristori, B. Schneider, E. Sexton-Kennedy, N. Smith, A. Soha, W. J. Spalding, L. Spiegel, S. Stoynev, J. Strait, L. Taylor, S. Tkaczyk, N. V. Tran, L. Uplegger, E. W. Vaandering, H. A. Weber, A. Woodard, D. Acosta, P. Avery, D. Bourilkov, L. Cadamuro, V. Cherepanov, F. Errico, R. D. Field, D. Guerrero, B. M. Joshi, M. Kim, J. Konigsberg, A. Korytov, K. H. Lo, K. Matchev, N. Menendez, G. Mitselmakher, D. Rosenzweig, K. Shi, J. Wang, S. Wang, X. Zuo, T. Adams, A. Askew, D. Diaz, R. Habibullah, S. Hagopian, V. Hagopian, K. F. Johnson, R. Khurana, T. Kolberg, G. Martinez, H. Prosper, C. Schiber, R. Yohay, J. Zhang, M. M. Baarmand, S. Butalla, T. Elkafrawy, M. Hohlmann, D. Noonan, M. Rahmani, M. Saunders, F. Yumiceva, M. R. Adams, L. Apanasevich, H. Becerril Gonzalez, R. Cavanaugh, X. Chen, S. Dittmer, O. Evdokimov, C. E. Gerber, D. A. Hangal, D. J. Hofman, C. Mills, G. Oh, T. Roy, M. B. Tonjes, N. Varelas, J. Viinikainen, X. Wang, Z. Wu, M. Alhusseini, K. Dilsiz, S. Durgut, R. P. Gandrajula, M. Haytmyradov, V. Khristenko, O. K. Köseyan, J.-P. Merlo, A. Mestvirishvili, A. Moeller, J. Nachtman, H. Ogul, Y. Onel, F. Ozok, A. Penzo, C. Snyder, E. Tiras, J. Wetzel, K. Yi, O. Amram, B. Blumenfeld, L. Corcodilos, M. Eminizer, A. V. Gritsan, S. Kyriacou, P. Maksimovic, C. Mantilla, J. Roskes, M. Swartz, T.Á. Vámi, C. Baldenegro Barrera, P. Baringer, A. Bean, A. Bylinkin, T. Isidori, S. Khalil, J. King, G. Krintiras, A. Kropivnitskaya, C. Lindsey, N. Minafra, M. Murray, C. Rogan, C. Royon, S. Sanders, E. Schmitz, J. D. Tapia Takaki, Q. Wang, J. Williams, G. Wilson, S. Duric, A. Ivanov, K. Kaadze, D. Kim, Y. Maravin, T. Mitchell, A. Modak, A. Mohammadi, F. Rebassoo, D. Wright, E. Adams, A. Baden, O. Baron, A. Belloni, S. C. Eno, Y. Feng, N. J. Hadley, S. Jabeen, G. Y. Jeng, R. G. Kellogg, T. Koeth, A. C. Mignerey, S. Nabili, M. Seidel, A. Skuja, S. C. Tonwar, L. Wang, K. Wong, D. Abercrombie, B. Allen, R. Bi, S. Brandt, W. Busza, I. A. Cali, Y. Chen, M. D’Alfonso, G. Gomez Ceballos, M. Goncharov, P. Harris, D. Hsu, M. Hu, M. Klute, D. Kovalskyi, J. Krupa, Y.-J. Lee, P. D. Luckey, B. Maier, A. C. Marini, C. Mcginn, C. Mironov, S. Narayanan, X. Niu, C. Paus, D. Rankin, C. Roland, G. Roland, Z. Shi, G. S. F. Stephans, K. Sumorok, K. Tatar, D. Velicanu, J. Wang, T. W. Wang, Z. Wang, B. Wyslouch, R. M. Chatterjee, A. Evans, S. Guts, P. Hansen, J. Hiltbrand, Sh. Jain, M. Krohn, Y. Kubota, Z. Lesko, J. Mans, M. Revering, R. Rusack, R. Saradhy, N. Schroeder, N. Strobbe, M. A. Wadud, J. G. Acosta, S. Oliveros, K. Bloom, S. Chauhan, D. R. Claes, C. Fangmeier, L. Finco, F. Golf, J. R. González Fernández, I. Kravchenko, J. E. Siado, G. R. Snow, B. Stieger, W. Tabb, F. Yan, G. Agarwal, H. Bandyopadhyay, C. Harrington, L. Hay, I. Iashvili, A. Kharchilava, C. McLean, D. Nguyen, J. Pekkanen, S. Rappoccio, B. Roozbahani, G. Alverson, E. Barberis, C. Freer, Y. Haddad, A. Hortiangtham, J. Li, G. Madigan, B. Marzocchi, D. M. Morse, V. Nguyen, T. Orimoto, A. Parker, L. Skinnari, A. Tishelman-Charny, T. Wamorkar, B. Wang, A. Wisecarver, D. Wood, S. Bhattacharya, J. Bueghly, Z. Chen, A. Gilbert, T. Gunter, K. A. Hahn, N. Odell, M. H. Schmitt, K. Sung, M. Velasco, R. Bucci, N. Dev, R. Goldouzian, M. Hildreth, K. Hurtado Anampa, C. Jessop, D. J. Karmgard, K. Lannon, W. Li, N. Loukas, N. Marinelli, I. Mcalister, F. Meng, K. Mohrman, Y. Musienko, R. Ruchti, P. Siddireddy, S. Taroni, M. Wayne, A. Wightman, M. Wolf, L. Zygala, J. Alimena, B. Bylsma, B. Cardwell, L. S. Durkin, B. Francis, C. Hill, A. Lefeld, B. L. Winer, B. R. Yates, P. Das, G. Dezoort, P. Elmer, B. Greenberg, N. Haubrich, S. Higginbotham, A. Kalogeropoulos, G. Kopp, S. Kwan, D. Lange, M. T. Lucchini, J. Luo, D. Marlow, K. Mei, I. Ojalvo, J. Olsen, C. Palmer, P. Piroué, D. Stickland, C. Tully, S. Malik, S. Norberg, V. E. Barnes, R. Chawla, S. Das, L. Gutay, M. Jones, A. W. Jung, B. Mahakud, G. Negro, N. Neumeister, C. C. Peng, S. Piperov, H. Qiu, J. F. Schulte, M. Stojanovic, N. Trevisani, F. Wang, R. Xiao, W. Xie, T. Cheng, J. Dolen, N. Parashar, A. Baty, S. Dildick, K. M. Ecklund, S. Freed, F. J. M. Geurts, M. Kilpatrick, A. Kumar, W. Li, B. P. Padley, R. Redjimi, J. Roberts, J. Rorie, W. Shi, A. G. Stahl Leiton, A. Bodek, P. de Barbaro, R. Demina, J. L. Dulemba, C. Fallon, T. Ferbel, M. Galanti, A. Garcia-Bellido, O. Hindrichs, A. Khukhunaishvili, E. Ranken, R. Taus, B. Chiarito, J. P. Chou, A. Gandrakota, Y. Gershtein, E. Halkiadakis, A. Hart, M. Heindl, E. Hughes, S. Kaplan, O. Karacheban, I. Laflotte, A. Lath, R. Montalvo, K. Nash, M. Osherson, S. Salur, S. Schnetzer, S. Somalwar, R. Stone, S. A. Thayil, S. Thomas, H. Wang, H. Acharya, A. G. Delannoy, S. Spanier, O. Bouhali, M. Dalchenko, A. Delgado, R. Eusebi, J. Gilmore, T. Huang, T. Kamon, H. Kim, S. Luo, S. Malhotra, R. Mueller, D. Overton, L. Perniè, D. Rathjens, A. Safonov, J. Sturdy, N. Akchurin, J. Damgov, V. Hegde, S. Kunori, K. Lamichhane, S. W. Lee, T. Mengke, S. Muthumuni, T. Peltola, S. Undleeb, I. Volobouev, Z. Wang, A. Whitbeck, E. Appelt, S. Greene, A. Gurrola, R. Janjam, W. Johns, C. Maguire, A. Melo, H. Ni, K. Padeken, F. Romeo, P. Sheldon, S. Tuo, J. Velkovska, M. Verweij, M. W. Arenton, B. Cox, G. Cummings, J. Hakala, R. Hirosky, M. Joyce, A. Ledovskoy, A. Li, C. Neu, B. Tannenwald, Y. Wang, E. Wolfe, F. Xia, P. E. Karchin, N. Poudyal, P. Thapa, K. Black, T. Bose, J. Buchanan, C. Caillol, S. Dasu, I. De Bruyn, P. Everaerts, C. Galloni, H. He, M. Herndon, A. Hervé, U. Hussain, A. Lanaro, A. Loeliger, R. Loveless, J. Madhusudanan Sreekala, A. Mallampalli, D. Pinna, T. Ruggles, A. Savin, V. Shang, V. Sharma, W. H. Smith, D. Teague, S. Trembath-reichert, W. Vetens, CMS Collaboration

**Affiliations:** 1 Yerevan Physics Institute, Yerevan, Armenia; 2 Institut für Hochenergiephysik, Vienna, Austria; 3 Institute for Nuclear Problems, Minsk, Belarus; 4 Universiteit Antwerpen, Antwerp, Belgium; 5 Vrije Universiteit Brussel, Brussels, Belgium; 6 Université Libre de Bruxelles, Brussels, Belgium; 7 Ghent University, Ghent, Belgium; 8 Université Catholique de Louvain, Louvain-la-Neuve, Belgium; 9 Centro Brasileiro de Pesquisas Fisicas, Rio de Janeiro, Brazil; 10 Universidade do Estado do Rio de Janeiro, Rio de Janeiro, Brazil; 11 Universidade Estadual Paulista, Universidade Federal do ABC, São Paulo, Brazil; 12 Institute for Nuclear Research and Nuclear Energy, Bulgarian Academy of Sciences, Sofia, Bulgaria; 13 University of Sofia, Sofia, Bulgaria; 14 Beihang University, Beijing, China; 15 Department of Physics, Tsinghua University, Beijing, China; 16 Institute of High Energy Physics, Beijing, China; 17 State Key Laboratory of Nuclear Physics and Technology, Peking University, Beijing, China; 18 Sun Yat-Sen University, Guangzhou, China; 19 Institute of Modern Physics and Key Laboratory of Nuclear Physics and Ion-beam Application (MOE), Fudan University, Shanghai, China; 20 Zhejiang University, Hangzhou, China; 21 Universidad de Los Andes, Bogota, Colombia; 22 Universidad de Antioquia, Medellin, Colombia; 23 University of Split, Faculty of Electrical Engineering, Mechanical Engineering and Naval Architecture, Split, Croatia; 24 University of Split, Faculty of Science, Split, Croatia; 25 Institute Rudjer Boskovic, Zagreb, Croatia; 26 University of Cyprus, Nicosia, Cyprus; 27 Charles University, Prague, Czech Republic; 28 Escuela Politecnica Nacional, Quito, Ecuador; 29 Universidad San Francisco de Quito, Quito, Ecuador; 30 Academy of Scientific Research and Technology of the Arab Republic of Egypt, Egyptian Network of High Energy Physics, Cairo, Egypt; 31 Center for High Energy Physics (CHEP-FU), Fayoum University, El-Fayoum, Egypt; 32 National Institute of Chemical Physics and Biophysics, Tallinn, Estonia; 33 Department of Physics, University of Helsinki, Helsinki, Finland; 34 Helsinki Institute of Physics, Helsinki, Finland; 35 Lappeenranta University of Technology, Lappeenranta, Finland; 36 IRFU, CEA, Université Paris-Saclay, Gif-sur-Yvette, France; 37 Laboratoire Leprince-Ringuet, CNRS/IN2P3, Ecole Polytechnique, Institut Polytechnique de Paris, Paris, France; 38 Université de Strasbourg, CNRS, IPHC UMR 7178, Strasbourg, France; 39 Université de Lyon, Université Claude Bernard Lyon 1, CNRS-IN2P3, Institut de Physique Nucléaire de Lyon, Villeurbanne, France; 40 Georgian Technical University, Tbilisi, Georgia; 41 RWTH Aachen University, I. Physikalisches Institut, Aachen, Germany; 42 RWTH Aachen University, III. Physikalisches Institut A, Aachen, Germany; 43 RWTH Aachen University, III. Physikalisches Institut B, Aachen, Germany; 44 Deutsches Elektronen-Synchrotron, Hamburg, Germany; 45 University of Hamburg, Hamburg, Germany; 46 Karlsruher Institut fuer Technologie, Karlsruhe, Germany; 47 Institute of Nuclear and Particle Physics (INPP), NCSR Demokritos, Aghia Paraskevi, Greece; 48 National and Kapodistrian University of Athens, Athens, Greece; 49 National Technical University of Athens, Athens, Greece; 50 University of Ioánnina, Ioánnina, Greece; 51 MTA-ELTE Lendület CMS Particle and Nuclear Physics Group, Eötvös Loránd University, Budapest, Hungary; 52 Wigner Research Centre for Physics, Budapest, Hungary; 53 Institute of Nuclear Research ATOMKI, Debrecen, Hungary; 54 Institute of Physics, University of Debrecen, Debrecen, Hungary; 55 Eszterhazy Karoly University, Karoly Robert Campus, Gyongyos, Hungary; 56 Indian Institute of Science (IISc), Bangalore, India; 57 National Institute of Science Education and Research, HBNI, Bhubaneswar, India; 58 Panjab University, Chandigarh, India; 59 University of Delhi, Delhi, India; 60 Saha Institute of Nuclear Physics, HBNI, Kolkata, India; 61 Indian Institute of Technology Madras, Madras, India; 62 Bhabha Atomic Research Centre, Mumbai, India; 63 Tata Institute of Fundamental Research-A, Mumbai, India; 64 Tata Institute of Fundamental Research-B, Mumbai, India; 65 Indian Institute of Science Education and Research (IISER), Pune, India; 66 Department of Physics, Isfahan University of Technology, Isfahan, Iran; 67 Institute for Research in Fundamental Sciences (IPM), Tehran, Iran; 68 University College Dublin, Dublin, Ireland; 69 INFN Sezione di Bari , Università di Bari, Politecnico di Bari, Bari, Italy; 70 INFN Sezione di Bologna, Università di Bologna, Bologna, Italy; 71 INFN Sezione di Catania, Università di Catania, Catania, Italy; 72 INFN Sezione di Firenze, Università di Firenze, Florence, Italy; 73 INFN Laboratori Nazionali di Frascati, Frascati, Italy; 74 INFN Sezione di Genova, Università di Genova, Genoa, Italy; 75 INFN Sezione di Milano-Bicocca, Università di Milano-Bicocca, Milan, Italy; 76 INFN Sezione di Napoli , Università di Napoli ’Federico II’ , Napoli, Italy, Università della Basilicata , Potenza, Italy, Università G. Marconi, Rome, Italy; 77 INFN Sezione di Padova , Università di Padova , Padova, Italy, Università di Trento, Trento, Italy; 78 INFN Sezione di Pavia, Università di Pavia, Pavia, Italy; 79 INFN Sezione di Perugia, Università di Perugia, Perugia, Italy; 80 INFN Sezione di Pisa , Università di Pisa, Scuola Normale Superiore di Pisa, Pisa, Italy; 81 INFN Sezione di Roma, Sapienza Università di Roma, Rome, Italy; 82 INFN Sezione di Torino , Università di Torino , Turin, Italy, Università del Piemonte Orientale, Novara, Italy; 83 INFN Sezione di Trieste, Università di Trieste, Trieste, Italy; 84 Kyungpook National University, Daegu, Korea; 85 Chonnam National University, Institute for Universe and Elementary Particles, Kwangju, Korea; 86 Hanyang University, Seoul, Korea; 87 Korea University, Seoul, Korea; 88 Kyung Hee University, Department of Physics, Seoul, Republic of Korea; 89 Sejong University, Seoul, Korea; 90 Seoul National University, Seoul, Korea; 91 University of Seoul, Seoul, Korea; 92 Department of Physics, Yonsei University, Seoul, Korea; 93 Sungkyunkwan University, Suwon, Korea; 94 Riga Technical University, Riga, Latvia; 95 Vilnius University, Vilnius, Lithuania; 96 National Centre for Particle Physics, Universiti Malaya, Kuala Lumpur, Malaysia; 97 Universidad de Sonora (UNISON), Hermosillo, Mexico; 98 Centro de Investigacion y de Estudios Avanzados del IPN, Mexico City, Mexico; 99 Universidad Iberoamericana, Mexico City, Mexico; 100 Benemerita Universidad Autonoma de Puebla, Puebla, Mexico; 101 Universidad Autónoma de San Luis Potosí, San Luis Potosí, Mexico; 102 University of Montenegro, Podgorica, Montenegro; 103 University of Auckland, Auckland, New Zealand; 104 University of Canterbury, Christchurch, New Zealand; 105 National Centre for Physics, Quaid-I-Azam University, Islamabad, Pakistan; 106 AGH University of Science and Technology, Faculty of Computer Science, Electronics and Telecommunications, Krakow, Poland; 107 National Centre for Nuclear Research, Swierk, Poland; 108 Institute of Experimental Physics, Faculty of Physics, University of Warsaw, Warsaw, Poland; 109 Laboratório de Instrumentação e Física Experimental de Partículas, Lisbon, Portugal; 110 Joint Institute for Nuclear Research, Dubna, Russia; 111 Petersburg Nuclear Physics Institute, Gatchina (St. Petersburg), Russia; 112 Institute for Nuclear Research, Moscow, Russia; 113 Institute for Theoretical and Experimental Physics named by A.I. Alikhanov of NRC ‘Kurchatov Institute’, Moscow, Russia; 114 Moscow Institute of Physics and Technology, Moscow, Russia; 115 National Research Nuclear University ’Moscow Engineering Physics Institute’ (MEPhI), Moscow, Russia; 116 P.N. Lebedev Physical Institute, Moscow, Russia; 117 Skobeltsyn Institute of Nuclear Physics, Lomonosov Moscow State University, Moscow, Russia; 118 Novosibirsk State University (NSU), Novosibirsk, Russia; 119 Institute for High Energy Physics of National Research Centre ‘Kurchatov Institute’, Protvino, Russia; 120 National Research Tomsk Polytechnic University, Tomsk, Russia; 121 Tomsk State University, Tomsk, Russia; 122 University of Belgrade, Faculty of Physics and VINCA Institute of Nuclear Sciences, Belgrade, Serbia; 123 Centro de Investigaciones Energéticas Medioambientales y Tecnológicas (CIEMAT), Madrid, Spain; 124 Universidad Autónoma de Madrid, Madrid, Spain; 125 Universidad de Oviedo, Instituto Universitario de Ciencias y Tecnologías Espaciales de Asturias (ICTEA), Oviedo, Spain; 126 Instituto de Física de Cantabria (IFCA), CSIC-Universidad de Cantabria, Santander, Spain; 127 University of Colombo, Colombo, Sri Lanka; 128 Department of Physics, University of Ruhuna, Matara, Sri Lanka; 129 CERN, European Organization for Nuclear Research, Geneva, Switzerland; 130 Paul Scherrer Institut, Villigen, Switzerland; 131 ETH Zurich, Institute for Particle Physics and Astrophysics (IPA), Zurich, Switzerland; 132 Universität Zürich, Zurich, Switzerland; 133 National Central University, Chung-Li, Taiwan; 134 National Taiwan University (NTU), Taipei, Taiwan; 135 Department of Physics, Faculty of Science, Chulalongkorn University, Bangkok, Thailand; 136 Physics Department, Science and Art Faculty, Çukurova University, Adana, Turkey; 137 Physics Department, Middle East Technical University, Ankara, Turkey; 138 Bogazici University, Istanbul, Turkey; 139 Istanbul Technical University, Istanbul, Turkey; 140 Istanbul University, Istanbul, Turkey; 141 Institute for Scintillation Materials of National Academy of Science of Ukraine, Kharkov, Ukraine; 142 National Scientific Center, Kharkov Institute of Physics and Technology, Kharkov, Ukraine; 143 University of Bristol, Bristol, UK; 144 Rutherford Appleton Laboratory, Didcot, UK; 145 Imperial College, London, UK; 146 Brunel University, Uxbridge, UK; 147 Baylor University, Waco, USA; 148 Catholic University of America, Washington, DC USA; 149 The University of Alabama, Tuscaloosa, USA; 150 Boston University, Boston, USA; 151 Brown University, Providence, USA; 152 University of California, Davis, Davis, USA; 153 University of California, Los Angeles, USA; 154 University of California, Riverside, Riverside, USA; 155 University of California, San Diego, La Jolla, USA; 156 Department of Physics, University of California, Santa Barbara, Santa Barbara, USA; 157 California Institute of Technology, Pasadena, USA; 158 Carnegie Mellon University, Pittsburgh, USA; 159 University of Colorado Boulder, Boulder, USA; 160 Cornell University, Ithaca, USA; 161 Fermi National Accelerator Laboratory, Batavia, USA; 162 University of Florida, Gainesville, USA; 163 Florida State University, Tallahassee, USA; 164 Florida Institute of Technology, Melbourne, USA; 165 University of Illinois at Chicago (UIC), Chicago, USA; 166 The University of Iowa, Iowa City, USA; 167 Johns Hopkins University, Baltimore, USA; 168 The University of Kansas, Lawrence, USA; 169 Kansas State University, Manhattan, USA; 170 Lawrence Livermore National Laboratory, Livermore, USA; 171 University of Maryland, College Park, USA; 172 Massachusetts Institute of Technology, Cambridge, USA; 173 University of Minnesota, Minneapolis, USA; 174 University of Mississippi, Oxford, USA; 175 University of Nebraska-Lincoln, Lincoln, USA; 176 State University of New York at Buffalo, Buffalo, USA; 177 Northeastern University, Boston, USA; 178 Northwestern University, Evanston, USA; 179 University of Notre Dame, Notre Dame, USA; 180 The Ohio State University, Columbus, USA; 181 Princeton University, Princeton, USA; 182 University of Puerto Rico, Mayaguez, USA; 183 Purdue University, West Lafayette, USA; 184 Purdue University Northwest, Hammond, USA; 185 Rice University, Houston, USA; 186 University of Rochester, Rochester, USA; 187 Rutgers, The State University of New Jersey, Piscataway, USA; 188 University of Tennessee, Knoxville, USA; 189 Texas A&M University, College Station, USA; 190 Texas Tech University, Lubbock, USA; 191 Vanderbilt University, Nashville, USA; 192 University of Virginia, Charlottesville, USA; 193 Wayne State University, Detroit, USA; 194 University of Wisconsin-Madison, Madison, WI USA; 195 CERN, 1211 Geneva 23, Switzerland

## Abstract

A search is presented for supersymmetric partners of the top quark (top squarks) in final states with two oppositely charged leptons (electrons or muons), jets identified as originating from ${\text {b}}$quarks, and missing transverse momentum. The search uses data from proton-proton collisions at $\sqrt{s}=13\,\text {TeV}$ collected with the CMS detector, corresponding to an integrated luminosity of 137$\,{\text {fb}}^{-1}$. Hypothetical signal events are efficiently separated from the dominant top quark pair production background with requirements on the significance of the missing transverse momentum and on transverse mass variables. No significant deviation is observed from the expected background. Exclusion limits are set in the context of simplified supersymmetric models with pair-produced lightest top squarks. For top squarks decaying exclusively to a top quark and a lightest neutralino, lower limits are placed at $95\%$ confidence level on the masses of the top squark and the neutralino up to 925 and 450$\,\text {GeV}$, respectively. If the decay proceeds via an intermediate chargino, the corresponding lower limits on the mass of the lightest top squark are set up to 850$\,\text {GeV}$ for neutralino masses below 420$\,\text {GeV}$. For top squarks undergoing a cascade decay through charginos and sleptons, the mass limits reach up to 1.4$\,\text {TeV}$ and 900$\,\text {GeV}$ respectively for the top squark and the lightest neutralino.

## Introduction

The standard model (SM) of particle physics accurately describes the overwhelming majority of observed particle physics phenomena. Nevertheless, several open questions are not addressed by the SM, such as the hierarchy problem, the need for fine tuning to reconcile the large difference between the electroweak and the Planck scales in the presence of a fundamental scalar [1–4]. Moreover, there is a lack of an SM candidate particle that could constitute the dark matter in cosmological and astrophysical observations [5, 6]. Supersymmetry (SUSY) [7–14] is a well-motivated extension of the SM that provides a solution to both of these problems, through the introduction of a symmetry between bosons and fermions. In SUSY models, large quantum loop corrections to the mass of the Higgs boson (H), mainly arising from the top quarks, are mostly canceled by those arising from their SUSY partners, the top squarks, if the masses of the SM particles and their SUSY partners are close in value. Similar cancellations occur for other particles, resulting in a natural solution to the hierarchy problem [2, 15, 16]. Furthermore, SUSY introduces a new quantum number, R parity [17], that distinguishes between SUSY and SM particles. If R parity is conserved, top squarks are produced in pairs and the lightest SUSY particle (LSP) is stable. If neutral, the LSP provides a good candidate for the dark matter. The lighter top squark mass eigenstate $\tilde{{\text {t}}}_{1}$ is the lightest squark in many SUSY models and may be within the energy reach of the CERN LHC if SUSY provides a natural solution to the hierarchy problem [18]. This strongly motivates searches for top squark production.

In this paper, we present a search for top squark pair production in data from proton-proton (${\text {p}}{\text {p}}$) collisions collected at a center-of-mass energy of 13$\,\text {TeV}$, corresponding to an integrated luminosity of 137$\,{\text {fb}}^{-1}$, with the CMS detector at the LHC from 2016 to 2018. The search is performed in final states with two leptons (electrons or muons), hadronic jets identified as originating from ${\text {b}}$quarks, and significant missing transverse momentum ($p_{\mathrm {T}} ^{\text {miss}}$). The large background from the SM top quark–antiquark pair production ($\hbox {t}{\bar{\hbox {t}}}$) is reduced by several orders of magnitude through the use of specially designed transverse-mass variables [19, 20]. Simulations of residual SM backgrounds in the search regions are validated in control regions orthogonal to the signal regions, using observed data.

**Fig. 1:**
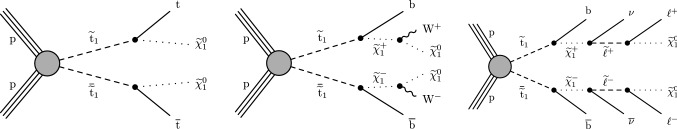
Diagrams for simplified SUSY models with strong production of top squark pairs $\tilde{{\text {t}}}_{1} \overline{\widetilde{\tilde{{\text {t}}}}_{1}} _{1}$. In the $\text {T2}{\text {t}}{\text {t}}$ model (left), the top squark decays to a top quark and a ${\tilde{\chi }}^{0}_{1}$. In the $\text {T2}{{\text {b}}{\text {W}}}$ model (center), the top squark decays into a bottom quark and an intermediate ${\tilde{\chi }}^\pm _{1}$ that further decays into a ${\text {W}}$boson and a ${\tilde{\chi }}^{0}_{1}$. The decay of the intermediate ${\tilde{\chi }}^\pm _{1}$, which yields a $\nu$, plus a ${\tilde{\chi }}^{0}_{1}$ and a $\ell ^\pm$ from the decay of an intermediate slepton ${\widetilde{\ell }}^{\pm }$, is described by the $\text {T8}{\text {b}}{\text {b}}\ell \ell \nu \nu$ model (right)

Simplified models [21–23] of strong top squark pair production and different top squark decay modes are considered. Following the naming convention in Ref. [24], top squark decays to top quarks and neutralinos (${\tilde{\chi }}^{0}_{1}$, identified as LSPs) are described by the $\text {T2}{\text {t}}{\text {t}}$ model (Fig. 1, left). In the $\text {T2}{{\text {b}}{\text {W}}}$ model (Fig. 1, center), both top squarks decay via an intermediate chargino (${\tilde{\chi }}^\pm _{1}$) into a bottom quark, a ${\text {W}}$ boson, and an LSP. In both models, the undetected LSPs and the neutrinos from leptonic ${\text {W}}$decays account for significant $p_{\mathrm {T}} ^{\text {miss}}$, and the leptons provide a final state with low SM backgrounds. In the $\text {T8}{\text {b}}{\text {b}}\ell \ell \nu \nu$ model (Fig. 1, right), both top squarks decay via an intermediate chargino to a bottom quark, a slepton, and a neutrino. The branching fraction of the chargino to sleptons is assumed to be identical for the three slepton flavors. The subsequent decay of the sleptons to neutralinos and leptons leads to a final state with the same particle content as in the $\text {T2}{\text {t}}{\text {t}}$ model, albeit without the suppression of the dilepton final state from the leptonic ${\text {W}}$boson branching fraction.

Searches for top squark production have been performed by the ATLAS [25–32] and CMS [33–40] Collaborations using 8 and 13$\,\text {TeV}$ ${\text {p}}{\text {p}}$ collision data. These searches disfavor top squark masses below about 1.1–1.3$\,\text {TeV}$ in a wide variety of production and decay scenarios. Here we present a search for top squark pair production in dilepton final states. With respect to a previous search in this final state [38], improved methods to suppress and estimate backgrounds from SM processes and a factor of about four larger data set increase the expected sensitivity by about 125$\,\text {GeV}$ in the $\tilde{{\text {t}}}_{1}$ mass. This search complements recent searches for top squark production in other final states [39, 40], in particular in scenarios with a compressed mass spectrum or final states with a single lepton.

## The CMS detector

The central feature of the CMS apparatus is a superconducting solenoid of 6$\,{\text {m}}$ internal diameter, providing a magnetic field of 3.8$\,{\text {T}}$. Within the solenoid volume are a silicon pixel and strip tracker, a lead tungstate crystal electromagnetic calorimeter (ECAL), and a brass and scintillator hadron calorimeter, each composed of a barrel and two endcap sections. Forward calorimeters extend the pseudorapidity ($\eta$) coverage provided by the barrel and endcap detectors that improve the measurement of the imbalance in transverse momentum. Muons are detected in gas-ionization chambers embedded in the steel flux-return yoke outside the solenoid.

Events of interest are selected using a two-tier trigger system. The first level, composed of custom hardware processors, uses information from the calorimeters and muon detectors to select events in a fixed time interval of less than 4$\mu$ $\,{\text {s}}$. The second level, called the high-level trigger, further decreases the event rate from around 100$\,{\text {kHz}}$ to less than 1$\,{\text {kHz}}$ before data storage [41]. A more detailed description of the CMS detector, together with a definition of the coordinate system used and the relevant kinematic variables, can be found in Ref. [42].

## Event samples

The search is performed in a data set collected by the CMS experiment during the 2016–2018 LHC running periods. Events are selected online by different trigger algorithms that require the presence of one or two leptons (electrons or muons). The majority of events are selected with dilepton triggers. The thresholds of same-flavor (SF) dilepton triggers are 23$\,\text {GeV}$ (electron) or 17$\,\text {GeV}$ (muon) on the transverse momentum ($p_{\mathrm {T}}$) of the leading lepton, and 12$\,\text {GeV}$ (electron) or 8$\,\text {GeV}$ (muon) on the subleading lepton $p_{\mathrm {T}}$. Triggers for different-flavor (DF) dileptons have thresholds of 23$\,\text {GeV}$ on the leading lepton $p_{\mathrm {T}}$, and 12$\,\text {GeV}$ (electron) or 8$\,\text {GeV}$ (muon) on the subleading lepton $p_{\mathrm {T}}$. Single lepton triggers with a 24$\,\text {GeV}$ threshold for muons and with a 27$\,\text {GeV}$ threshold for electrons (32$\,\text {GeV}$ for electrons in the years 2017 and 2018) improve the selection efficiency. The efficiency of this online selection is measured using observed events that are independently selected based on the presence of jets and requirements on the $p_{\mathrm {T}} ^{\text {miss}}$. Typical efficiencies range from 95 to 99%, depending on the $p_{\mathrm {T}}$ and $\eta$ of the two leptons and are accounted for by corrections applied to simulated events.

**Table 1:** Event generator and orders of accuracy for each simulated background process

Process	Cross section	Event generator	Perturbative
	normalization		order
$\hbox {t}{\bar{\hbox {t}}}$, single ${\text {t}}$	NNLO+NNLL	powheg v2	NLO
${\text {t}}{\text {W}}$	NNLO	powheg v1/v2	NLO
t$\bar{\mathrm{t}}$H	NLO+NLL	powheg v2	NLO
Drell–Yan	NNLO	MadGraph 5_amc@nlo	LO
$\hbox {t}{\bar{\hbox {t}}} {\text {Z}}$, $\hbox {t}{\bar{\hbox {t}}} {\text {W}}$, ${\text {t}}{\text {Z}}{\text {q}}$, $\hbox {t}{\bar{\hbox {t}}} {\upgamma }^{(*)}$,	NLO	MadGraph 5_amc@nlo	NLO
$\text {VVV}$, $\text {VV}$			
tHW, tHq	NLO	MadGraph 5_amc@nlo	LO
${\text {t}}{\text {W}}{\text {Z}}$	LO	MadGraph 5_amc@nlo	LO

Simulated samples matching the varying conditions for each data taking period are generated using Monte Carlo (MC) techniques. The $\hbox {t}{\bar{\hbox {t}}}$ production and *t*- and *s*-channel single-top-quark background processes are simulated at next-to-leading order (NLO) with the powheg v2 [43–50] event generator, and are normalized to next-to-next-to-leading-order (NNLO) cross sections, including soft-gluon resummation at next-to-next-to-leading-logarithmic (NNLL) accuracy [51]. Events with single top quarks produced in association with ${\text {W}}$bosons (${\text {t}}{\text {W}}$) are simulated with powheg v1 [52] (2016) or powheg v2 (2017–2018), and are normalized to the NNLO cross section [53, 54]. The $\hbox {t}{\bar{\hbox {t}}} \mathrm{H}$ process is generated with powheg v2 at NLO [55]. Drell–Yan events are generated with up to four extra partons in the matrix element calculations with MadGraph 5_amc@nlo v2.3.3 (2016) and v2.4.2 (2017–2018) [56] at leading order (LO), and the cross section is computed at NNLO [57]. The $\hbox {t}{\bar{\hbox {t}}} {\text {Z}}$, $\hbox {t}{\bar{\hbox {t}}} {\text {W}}$, ${\text {t}}{\text {Z}}{\text {q}}$, $\hbox {t}{\bar{\hbox {t}}} {\upgamma }^{(*)}$, and triboson ($\text {VVV}$) processes are generated with MadGraph 5_amc@nlo at NLO. The cross section of the $\hbox {t}{\bar{\hbox {t}}} {\text {Z}}$ process is computed at NLO in perturbative quantum chromodynamics (QCD) and electroweak accuracy [58, 59]. The $\hbox {t}{\bar{\hbox {t}}} \mathrm{H}$ process is normalized to a cross section calculated at NLO+NLL accuracy [60]. The diboson ($\text {VV}$) processes are simulated with up to one extra parton in the matrix element calculations, using MadGraph 5_amc@nlo at NLO. The ${\text {t}}{\text {W}}{\text {Z}}$, ${\text {t}}\mathrm{H}{\text {q}}$, and ${\text {t}}\mathrm{H}{\text {W}}$ processes are generated at LO with MadGraph 5_amc@nlo. These processes are normalized to the most precise available cross sections, corresponding to NLO accuracy in most cases. A summary of the event samples is provided in Table 1.

The event generators are interfaced with pythia v8.226 (8.230) [61] using the CUETP8M1 (CP5) tune [62–64] for 2016 (2017, 2018) samples to simulate the fragmentation, parton shower, and hadronization of partons in the initial and final states, along with the underlying event. The NNPDF parton distribution functions (PDFs) at different perturbative orders in QCD are used in v3.0 [65] and v3.1 [66] for 2016 and 2017–2018 samples, respectively. Double counting of the partons generated with MadGraph 5_amc@nlo and pythia is removed using the MLM [67] and the FxFx [68] matching schemes for LO and NLO samples, respectively. The events are subsequently processed with a Geant4-based simulation model [69] of the CMS detector.

The SUSY signal samples are generated with MadGraph 5_amc@nlo at LO precision, with up to two extra partons in the matrix element calculations, interfaced with pythia v8.226 (8.230) using the CUETP8M1 (CP2) tune for 2016 (2017, 2018). For the $\text {T2}{\text {t}}{\text {t}}$ and $\text {T2}{{\text {b}}{\text {W}}}$ models, the top squark mass is varied from 200 to 1200$\,\text {GeV}$ and the mass of the LSP is scanned from 1 to 650$\,\text {GeV}$. The mass of the chargino in the $\text {T2}{{\text {b}}{\text {W}}}$ model is assumed to be equal to the mean of the masses of the top squark and the lightest neutralino. For the $\text {T8}{\text {b}}{\text {b}}\ell \ell \nu \nu$ model, the top squark mass is varied from 200 to 1600$\,\text {GeV}$ and the mass of the LSP is scanned from 1 to 1200$\,\text {GeV}$. Similarly to the $\text {T2}{{\text {b}}{\text {W}}}$ model, the mass of the chargino is assumed to be equal to the mean of the top squark and the LSP masses. For the slepton mass, three values of $x = 0.95$, 0.50, 0.05 are chosen in $m_{{\widetilde{\ell }}} = x \, (m_{{\tilde{\chi }}^{+}_{1}} - m_{{\tilde{\chi }}^{0}_{1}}) + m_{{\tilde{\chi }}^{0}_{1}}$. The production cross sections of signal samples are normalized to approximate NNLO+NNLL accuracy with all other SUSY particles assumed to be heavy and decoupled [70–82]. The simulation of the detector response is performed using the CMS fast detector simulation [83, 84].

All simulated samples include the effects of additional ${\text {p}}{\text {p}}$ collisions in the same or adjacent bunch crossings (pileup), and are reweighted according to the observed distribution of the number of interactions per bunch crossing. An additional correction is applied to account for a mismatch of the simulated samples and the observed distribution of primary vertices in the 2018 running period.

## Object and event selection

Event reconstruction uses the CMS particle-flow (PF) algorithm [85], which provides an exclusive set of electron [86], muon [87], charged hadron, neutral hadron, and photon candidates. These particles are defined with respect to the primary ${\text {p}}{\text {p}}$ interaction vertex, which is the vertex with the largest value of summed physics-object $p_{\mathrm {T}} ^2$. The physics objects are the jets, clustered using the anti-$k_{{\mathrm {T}}}$ algorithm [88, 89] with the tracks assigned to candidate vertices as inputs, and the associated missing transverse momentum, taken as the negative vector sum of the $p_{\mathrm {T}}$ of those jets. Charged-hadron candidates not originating from the selected primary vertex in the event are discarded from the list of reconstructed particles.

Electron candidates are reconstructed using tracking and ECAL information, by combining the clusters of energy deposits in the ECAL with charged tracks [86]. The electron identification is performed using shower shape variables, track-cluster matching variables, and track quality variables. The selection is optimized to identify electrons from the decay of ${\text {W}}$and ${\text {Z}}$bosons while rejecting electron candidates originating from jets. To reject electrons originating from photon conversions inside the detector, electrons are required to have all possible measurements in the innermost tracker layers and to be incompatible with any conversion-like secondary vertices. Reconstruction of muon candidates is done by geometrically matching tracks from measurements in the muon system and tracker, and fitting them to form a global muon track. Muons are identified using the quality of the geometrical matching and the quality of the tracks [87].

In all three running periods, the selected lepton candidates are required to satisfy $p_{\mathrm {T}} > 30 \, (20)$ $\,\text {GeV}$ for the leading (subleading) lepton, and $|\eta | < 2.4$, and to be isolated. To obtain a measure of isolation for leptons with $p_{\mathrm {T}} <50\,\text {GeV}$, a cone with radius $\varDelta R=\sqrt{{(\varDelta \eta )}^2+{(\varDelta \phi )}^2}=0.2$ (where $\phi$ is the azimuthal angle in radians) is constructed around the lepton at the event vertex. For leptons with $p_{\mathrm {T}} >50\,\text {GeV}$ the radius is reduced to $\varDelta R=\mathrm {max}(0.05, 10\,\text {GeV}/p_{\mathrm {T}})$. A lepton is isolated if the scalar $p_{\mathrm {T}}$ sum of photons and neutral and charged hadrons reconstructed by the PF algorithm within this cone is less than 20% of the lepton $p_{\mathrm {T}}$, i.e. $I_{\text {rel}}<0.2$. The contribution of neutral particles from pileup interactions is estimated according to the method described in Ref. [86], and subtracted from the isolation sum. The remaining selection criteria applied to electrons, muons, and the reconstruction of jets and $p_{\mathrm {T}} ^{\text {miss}}$ are described in Ref. [38]. Jets are clustered from PF candidates using the anti-$k_{{\mathrm {T}}}$ algorithm with a distance parameter of $R=0.4$, and are required to satisfy $p_{\mathrm {T}} > 30\,\text {GeV}$, $|\eta | < 2.4$, and quality criteria. A multivariate ${\text {b}}$tagging discriminator algorithm, DeepCSV [90], is used to identify jets arising from ${\text {b}}$quark hadronization and decay (${\text {b}}$jets). The chosen working point has a mistag rate of approximately 1% for light-flavor jets and a corresponding ${\text {b}}$tagging efficiency of approximately 70%, depending on jet $p_{\mathrm {T}}$ and $\eta$.

Scale factors are applied to simulated events to take into account differences between the observed and simulated lepton reconstruction, identification, and isolation, and ${\text {b}}$tagging efficiencies. Typical corrections are less than 1% per lepton and less than 10% per ${\text {b}}$-tagged jet.

## Search strategy

We select events containing a pair of leptons with opposite charge. The invariant mass of the lepton pair $m(\ell \ell )$ is required to be greater than 20$\,\text {GeV}$ to suppress backgrounds with misidentified or nonprompt leptons from the hadronization of (heavy-flavor) jets in multijet events. Events with additional leptons with $p_{\mathrm {T}} > 15 \,\text {GeV}$ and satisfying a looser isolation criterion of $I_{\text {rel}}<0.4$ are rejected. Events with an SF lepton pair that is consistent with the SM Drell–Yan production are removed by requiring $|m_{{\text {Z}}} - m(\ell \ell ) | > 15\,\text {GeV}$, where $m_{{\text {Z}}}$ is the mass of the ${\text {Z}}$boson. To further suppress Drell–Yan and other vector boson backgrounds, we require the number of jets ($N_\text {jets}$) to be at least two and, among them, the number of ${\text {b}}$-tagged jets ($N_{{\text {b}}}$) to be at least one.

**Fig. 2:**
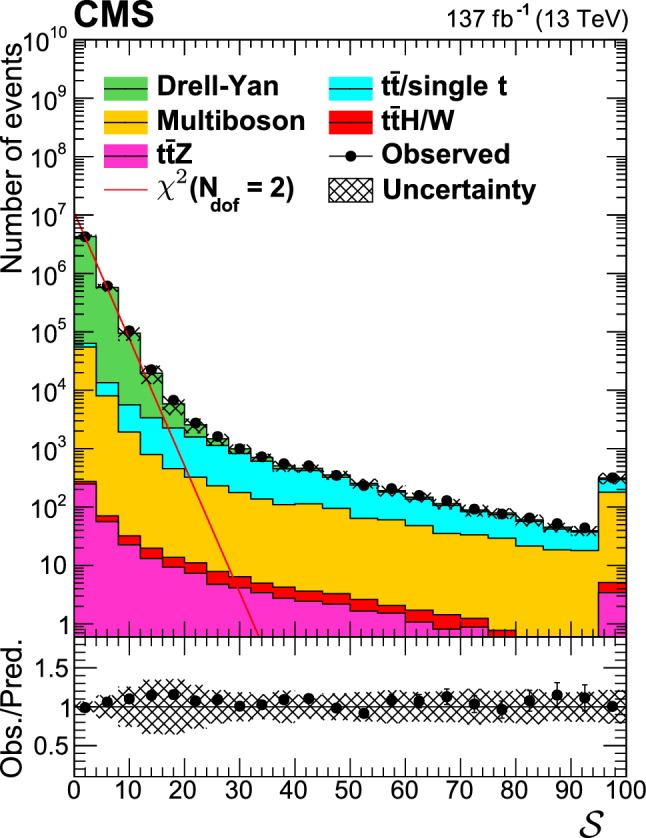
Distribution of $p_{\mathrm {T}} ^{\text {miss}}$ significance ${\mathcal {S}}$ in a ${\text {Z}}\rightarrow \ell \ell$ selection, requiring an SF lepton pair. Points with error bars represent the data, and the stacked histograms the SM backgrounds predicted as described in Sect. 6, with uncertainty in the SM prediction indicated by the hatched area. The red line represents a $\chi ^2$ distribution with two degrees of freedom. The last bin includes the overflow events. The lower panel gives the ratio between the observation and the predicted SM backgrounds. The relative uncertainty in the SM background prediction is shown as a hatched band

We use the $p_{\mathrm {T}} ^{\text {miss}}$ significance, denoted as ${\mathcal {S}}$, to suppress events where detector effects and misreconstruction of particles from pileup interactions are the main source of reconstructed $p_{\mathrm {T}} ^{\text {miss}}$. In short, the ${\mathcal {S}}$ observable offers an event-by-event assessment of the likelihood that the observed $p_{\mathrm {T}} ^{\text {miss}}$ is consistent with zero. Using a Gaussian parametrization of the resolutions of the reconstructed objects in the event, the ${\mathcal {S}}$ observable follows a $\chi ^2$-distribution with two degrees of freedom for events with no genuine $p_{\mathrm {T}} ^{\text {miss}}$ [91–93]. Figure 2 shows the distribution of ${\mathcal {S}}$ in a ${\text {Z}}\rightarrow \ell \ell$ sample, requiring events with two SF leptons with $|m_{{\text {Z}}} - m(\ell \ell ) | < 15\,\text {GeV}$, $N_\text {jets} \ge 2$ and $N_{{\text {b}}} =0$. Events with no genuine $p_{\mathrm {T}} ^{\text {miss}}$, such as from the Drell–Yan process, follow a $\chi ^2$ distribution with two degrees of freedom. Processes with true $p_{\mathrm {T}} ^{\text {miss}}$ such as $\hbox {t}{\bar{\hbox {t}}}$ or production of two or more ${\text {W}}$or ${\text {Z}}$bosons populate high values of the ${\mathcal {S}}$ distribution. The algorithm is described in Ref. [93] and provides stability of event selection efficiency as a function of the pileup rate. We exploit this property by requiring ${\mathcal {S}} >12$ in order to suppress the otherwise overwhelming Drell–Yan background in the SF channel. We further reduce this background by placing a requirement on the azimuthal angular separation of ${\vec {p}}_{{\mathrm {T}}}^{{\text {miss}}}$ and the momentum of the leading (subleading) jet of $\cos \varDelta \phi (p_{\mathrm {T}} ^{\text {miss}}, \mathrm {j})<0.80~(0.96)$. These criteria reject a small background of Drell–Yan events with significantly mismeasured jets.

The event preselection is summarized in Table 2. The resulting event sample is dominated by events with top quark pairs that decay to the dilepton final state.

**Table 2:** Overview of the event preselection requirements

Quantity	Requirement
$N_\mathrm {leptons}$	$=$ 2 (${\text {e}}$ or $\mu$), oppositely charged
$m(\ell \ell )$	> 20$\,\text {GeV}$
$|m_{{\text {Z}}} - m(\ell \ell ) |$	> 15$\,\text {GeV}$, SF only
$N_\text {jets}$	$\ge$ 2
$N_{{\text {b}}}$	$\ge$ 1
${\mathcal {S}}$	> 12
$\cos \varDelta \phi (p_{\mathrm {T}} ^{\text {miss}}, \mathrm {j}_{1})$	< 0.80
$\cos \varDelta \phi (p_{\mathrm {T}} ^{\text {miss}}, \mathrm {j}_{2})$	< 0.96

The main search variable in this analysis is [20, 94]

1 $$\begin{aligned} M_{\text {T2}}(\ell \ell )= & {} \min _{{\vec {p}}_{{\mathrm {T}}}^{{\text {miss}}} {}^{1} + {\vec {p}}_{{\mathrm {T}}}^{{\text {miss}}} {}^{2} = {\vec {p}}_{{\mathrm {T}}}^{{\text {miss}}}} \left( \max \left[ M_{\text {T}} \left( {\vec p}_{\mathrm {T}} ^{\text {vis}1},{\vec {p}}_{{\mathrm {T}}}^{{\text {miss}}} {}^{1}\right) ,\right. \right. \nonumber \\&\left. \left. M_{\text {T}} \left( {\vec p}_{\mathrm {T}} ^{\text {vis}2},{\vec {p}}_{{\mathrm {T}}}^{{\text {miss}}} {}^{2}\right) \right] \right) , \end{aligned}$$

where the choice ${\vec p}_{\mathrm {T}} ^{\text {vis}1,2}={\vec p}_{\mathrm {T}} ^{\ell 1,2}$ corresponds to the definition introduced in Ref. [95]. The alternative choice ${\vec p}_{\mathrm {T}} ^{\text {vis}1,2}={\vec p}_{\mathrm {T}} ^{\ell 1,2}+{\vec p}_{\mathrm {T}} ^{{\text {b}}1,2}$ involves the ${\text {b}}$-tagged jets and defines $M_{\text {T2}}({\text {b}}\ell {\text {b}}\ell )$. If only one ${\text {b}}$-tagged jet is found in the event, the jet with the highest $p_{\mathrm {T}}$ that does not pass the ${\text {b}}$ tagging selection is taken instead. The calculation of $M_{\text {T2}}(\ell \ell )$ and $M_{\text {T2}}({\text {b}}\ell {\text {b}}\ell )$ is performed through the algorithm discussed in Ref. [96], assuming vanishing mass for the undetected particles, and follows the description in Ref. [38]. The key feature of the $M_{\text {T2}}(\ell \ell )$ observable is that it retains a kinematic endpoint at the ${\text {W}}$boson mass for background events from the leptonic decays of two ${\text {W}}$bosons, produced directly or through top quark decay. Similarly, the $M_{\text {T2}}({\text {b}}\ell {\text {b}}\ell )$ observable is bound by the top quark mass if the leptons, neutrinos and ${\text {b}}$-tagged jets originate from the decay of top quarks. In turn, signal events from the processes depicted in Fig. 1 do not respect the endpoint and are expected to populate the tails of these distributions.

Signal regions based on $M_{\text {T2}}(\ell \ell )$, $M_{\text {T2}}({\text {b}}\ell {\text {b}}\ell )$ and ${\mathcal {S}}$ are defined to enhance sensitivity to different signal scenarios, and are listed in Table 3. The regions are further divided into different categories based on SF or DF lepton pairs, accounting for the different SM background composition. The signal regions are defined so that there is no overlap between them, nor with the background-enriched control regions.

**Table 3:** Definition of the signal regions. The regions are further split into SF and DF regions. The preselection in Table 2 is applied to all regions

$M_{\text {T2}}({\text {b}}\ell {\text {b}}\ell )$ ($\text {GeV}$)	${\mathcal {S}}$	$100< M_{\text {T2}}(\ell \ell ) < 140\,\text {GeV}$	$140< M_{\text {T2}}(\ell \ell ) < 240\,\text {GeV}$	$M_{\text {T2}}(\ell \ell ) > 240\,\text {GeV}$
0–100	12–50	SR0	SR6	
	> 50	SR1	SR7	
100–200	12–50	SR2	SR8	SR12
	> 50	SR3	SR9	
> 200	12–50	SR4	SR10	
	> 50	SR5	SR11	

## Background predictions

Events with an opposite-charge lepton pair are abundantly produced by Drell–Yan and $\hbox {t}{\bar{\hbox {t}}}$ processes. The event selection discussed in Sect. 4 efficiently rejects the vast majority of Drell–Yan events. Therefore, the major backgrounds from SM processes in the search regions are ${\text {t}}$/$\hbox {t}{\bar{\hbox {t}}}$ events that pass the $M_{\text {T2}}(\ell \ell )$ threshold because of severely mismeasured $p_{\mathrm {T}} ^{\text {miss}}$ or a misidentified lepton. In signal regions with large $M_{\text {T2}}(\ell \ell )$ and ${\mathcal {S}}$ requirements, $\hbox {t}{\bar{\hbox {t}}} {\text {Z}}$ events with ${\text {Z}}\rightarrow \nu \overline{\nu }$ are the main SM background. Remaining Drell–Yan events with large $p_{\mathrm {T}} ^{\text {miss}}$ from mismeasurement, multiboson production and other $\hbox {t}{\bar{\hbox {t}}}$/single ${\text {t}}$processes in association with a ${\text {W}}$, a ${\text {Z}}$or a Higgs boson ($\hbox {t}{\bar{\hbox {t}}} {\text {W}}$, ${\text {t}}{\text {q}} {\text {Z}}$ or $\hbox {t}{\bar{\hbox {t}}} \mathrm{H}$) are sources of smaller contributions. The background estimation procedures and their corresponding control regions, listed in Table 4, are discussed in the following.

**Table 4:** Definition of the control regions. The preselection in Table 2 is applied to all regions

Name	Definition	
TTCRSF	$M_{\text {T2}}(\ell \ell ) <100\,\text {GeV}$, SF leptons, $|m(\ell \ell )-m_{{\text {Z}}} |>15\,\text {GeV}$	
TTCRDF	$M_{\text {T2}}(\ell \ell ) <100\,\text {GeV}$, DF leptons	
TTZ2j2b		$N_\text {jets} = 2$, $N_{{\text {b}}} \ge 2$
TTZ3j1b	$N_{\ell }=3$, ${\mathcal {S}} \ge 0$, $\ge 1$ SF lepton pair	$N_\text {jets} = 3$, $N_{{\text {b}}} = 1$
TTZ3j2b	with $|m(\ell \ell )-m_{{\text {Z}}} |<10\,\text {GeV}$	$N_\text {jets} = 3$, $N_{{\text {b}}} \ge 2$
TTZ4j1b		$N_\text {jets} \ge 4$, $N_{{\text {b}}} = 1$
TTZ4j2b		$N_\text {jets} \ge 4$, $N_{{\text {b}}} \ge 2$
CR0-CR12	Same as SR0-SR12 in Table 3 but requiring SF leptons, $|m(\ell \ell )-m_{{\text {Z}}} |< 15\,\text {GeV}$,	
	$N_{{\text {b}}} = 0$, and without the $\cos \varDelta \phi (p_{\mathrm {T}} ^{\text {miss}}, \mathrm {j})$ requirements given in Table 2.	

### Top quark background

Events from the $\hbox {t}{\bar{\hbox {t}}}$ process are contained in the $M_{\text {T2}}(\ell \ell ) <100\,\text {GeV}$ region, as long as the jets and leptons in each event are identified and their momenta are precisely measured. Three main sources are identified that promote $\hbox {t}{\bar{\hbox {t}}}$ events into the tail of the $M_{\text {T2}}(\ell \ell )$ distribution. Firstly, the jet momentum resolution is approximately Gaussian [97] and jet mismeasurements propagate to $p_{\mathrm {T}} ^{\text {miss}}$, which subsequently leads to values of $M_{\text {T2}}(\ell \ell )$ and $M_{\text {T2}}({\text {b}}\ell {\text {b}}\ell )$ that do not obey the endpoint at the mother particle mass. For events with $M_{\text {T2}}(\ell \ell ) \le 140\,\text {GeV}$, this $\hbox {t}{\bar{\hbox {t}}}$ component is dominant, while it amounts to less than 10% for signal regions with $M_{\text {T2}}(\ell \ell ) >140\,\text {GeV}$. Secondly, significant mismeasurements of the momentum of jets can be caused by the loss of photons and neutral hadrons showering in masked channels of the calorimeters, or neutrinos with high $p_{\mathrm {T}}$ within jets. For $M_{\text {T2}}(\ell \ell ) >140\,\text {GeV}$, up to 50% of the top quark background falls into this category. The predicted rate and kinematic modeling of these rare non-Gaussian effects in simulation are checked in a control region requiring SF leptons satisfying $|m(\ell \ell )-m_{{\text {Z}}} |<15\,\text {GeV}$. A 30% uncertainty covers differences in the tails of the $p_{\mathrm {T}} ^{\text {miss}}$ distribution observed in this control region.

**Fig. 3:**
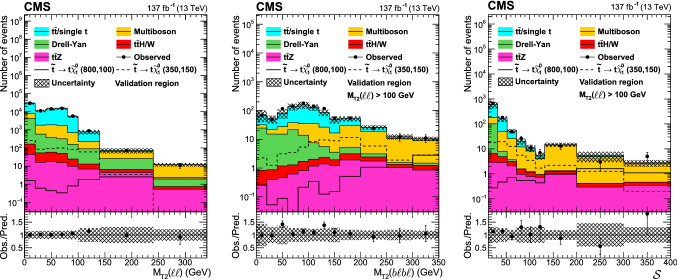
The $M_{\text {T2}}(\ell \ell )$, $M_{\text {T2}}({\text {b}}\ell {\text {b}}\ell )$, and ${\mathcal {S}}$ distributions in the validation regions requiring $N_\text {jets} \ge 2$ and $N_{{\text {b}}} =0$, combining the SF and DF channels. All other event selection requirements are applied. For the $M_{\text {T2}}({\text {b}}\ell {\text {b}}\ell )$ and ${\mathcal {S}}$ distributions, $M_{\text {T2}}(\ell \ell ) > 100\,\text {GeV}$ is required. The individual processes are scaled using their measured respective scale factors, as described in the text. The hatched band represents the experimental systematic uncertainties and the uncertainties in the scale factors. The last bin in each distribution includes the overflow events. The lower panel gives the ratio between the observation and the predicted SM backgrounds. The relative uncertainty in the SM background prediction is shown as a hatched band

Finally, an electron or a muon may fail the identification requirements, or the event may have a $\tau$ lepton produced in a ${\text {W}}$boson decay. If there is a nonprompt lepton from the hadronization of a bottom quark or a charged hadron misidentified as a lepton selected in the same event, the reconstructed value for $M_{\text {T2}}(\ell \ell )$ is not bound by the W mass. To validate the modeling of this contribution, we select events with one additional lepton satisfying loose isolation requirements on top of the selection in Table 2. In order to mimic the lost prompt-lepton background, we recompute $M_{\text {T2}}(\ell \ell )$ by combining each of the isolated leptons with the extra lepton in both the observed and simulated samples. Since the transverse momentum balance is not significantly changed by the lepton misidentification, the $p_{\mathrm {T}} ^{\text {miss}}$ and ${\mathcal {S}}$ observables are not modified. Events with misidentified electrons or muons from this category constitute up to 40% of the top quark background prediction for $M_{\text {T2}}(\ell \ell ) >140\,\text {GeV}$. We see good agreement between the observed and simulated kinematic distributions, indicating that the simulation describes such backgrounds well. Based on the statistical precision in the highest $M_{\text {T2}}(\ell \ell )$ regions, we assign a 50% uncertainty to this contribution.

The $\hbox {t}{\bar{\hbox {t}}}$ normalization is measured in situ by including a signal-depleted control region defined by $M_{\text {T2}}(\ell \ell ) <100\,\text {GeV}$ in the signal extraction fit, yielding a scale factor for the $\hbox {t}{\bar{\hbox {t}}}$ prediction of $1.02 \pm 0.04$. The region is split into DF (TTCRDF) and SF channels (TTCRSF). Events with a ${\text {Z}}$boson candidate are rejected in the latter.

**Table 5:** Typical values (90% quantiles) and maximum values of the systematic uncertainties in all signal regions

Systematic uncertainty	Typical (%)	Max (%)
Integrated luminosity	2	2
Pileup modeling	5	7
Jet energy scale	4	20
Jet energy resolution	3	4
${\text {b}}$ tagging efficiency	2	3
${\text {b}}$ tagging mistag rate	1	7
Trigger efficiency	1	2
Lepton identification efficiency	3	5
Modeling of unclustered energy	3	7
Non-Gaussian jet mismeasurements	6	6
Misidentified or nonprompt leptons	5	5
$\hbox {t}{\bar{\hbox {t}}}$ normalization	9	9
$\hbox {t}{\bar{\hbox {t}}} {\text {Z}}$ normalization	10	14
Multiboson background normalization	4	8
$\hbox {t}{\bar{\hbox {t}}} \mathrm{H}$/${\text {W}}$ background normalization	5	8
Drell–Yan normalization	3	8
Parton distribution functions	2	4
$\mu _\mathrm {R}$ and $\mu _\mathrm {F}$ choice	7	11

### Top quark + X background

Top quarks produced in association with a boson ($\hbox {t}{\bar{\hbox {t}}} {\text {Z}}$, $\hbox {t}{\bar{\hbox {t}}} {\text {W}}$, $\hbox {t}{\bar{\hbox {t}}} \mathrm{H}$, ${\text {t}}{\text {q}} {\text {Z}}$) form an irreducible background, if the boson decays to leptons or neutrinos. The ${\text {Z}}\rightarrow \nu \overline{\nu }$ decay in the $\hbox {t}{\bar{\hbox {t}}} {\text {Z}}$ process provides genuine $p_{\mathrm {T}} ^{\text {miss}}$ and is the dominant background component at high values of $M_{\text {T2}}(\ell \ell )$. The decay mode  is used to measure the normalization of this contribution. The leading, subleading, and trailing lepton $p_{\mathrm {T}}$ are required to satisfy thresholds of 40, 20, and $20\,\text {GeV}$, respectively. The invariant mass of two SF leptons with opposite charge is required to satisfy the tightened requirement $|m(\ell \ell ) - m_{{\text {Z}}} | <10\,\text {GeV}$. The shape of the distribution of $p_{\mathrm {T}} ({\text {Z}})$ has recently been measured in the 2016 and 2017 data sets [98] and is well described by simulation. Five control regions requiring different $N_\text {jets}$ and $N_{{\text {b}}}$ combinations are defined in Table 4 and labeled TTZ2j2b–TTZ4j2b. They are included in the signal extraction fit, in which the simulated number of $\hbox {t}{\bar{\hbox {t}}} {\text {Z}}$ events is found to be scaled up by a factor of $1.22 \pm 0.25$, consistent with the initial prediction.

### Drell–Yan and multiboson backgrounds

In order to measure the small residual Drell–Yan contribution that passes the event selection, we select dilepton events according to the criteria listed in Table 2 except that we invert the ${\text {Z}}$boson veto, the ${\text {b}}$jet requirements, and remove the angular separation requirements on jets and ${\vec {p}}_{{\mathrm {T}}}^{{\text {miss}}}$. We expect from the simulation that the selection is dominated by the Drell–Yan and multiboson events. For each SF signal region, we define a corresponding control region with the selections above and the signal region requirements on $M_{\text {T2}}(\ell \ell )$, $M_{\text {T2}}({\text {b}}\ell {\text {b}}\ell )$, and ${\mathcal {S}}$. The regions are labeled CR0–CR12 in Table 4 and are included in the signal extraction fit. The $M_{\text {T2}}({\text {b}}\ell {\text {b}}\ell )$ observable is calculated in these regions using the two highest $p_{\mathrm {T}}$ jets. The scale factors for the Drell–Yan and multiboson background components are found to be $1.18 \pm 0.28$ and $1.35 \pm 0.32$, respectively.

The good modeling of the multiboson and $\hbox {t}{\bar{\hbox {t}}}$ processes, including potential sources of anomalous $p_{\mathrm {T}} ^{\text {miss}}$, is demonstrated in a validation region requiring $N_\text {jets} \ge 2$ and $N_{{\text {b}}} =0$ and combining the SF and DF channels. The observed distributions of the search variables are compared with the simulated distributions in Fig. 3. The hatched band includes the experimental systematic uncertainties and the uncertainties in the background normalizations.

## Systematic uncertainties

Several experimental uncertainties affect the signal and background yield estimations. The efficiency of the trigger selection ranges from 95 to 99% with uncertainties lower than 2.3% in all signal and control regions. Offline lepton reconstruction and selection efficiencies are measured using ${\text {Z}}\rightarrow \ell \ell$ events in bins of lepton $p_{\mathrm {T}}$ and $\eta$. These measurements are performed separately in the observed and simulated data sets, with efficiency values ranging from 70 to 80%. Scale factors are used to correct the efficiencies measured in simulated events to those in the observed data. The uncertainties in these scale factors are less than 3% per lepton and less than 5% in most of the search and control regions.

Uncertainties in the event yields resulting from the calibration of the jet energy scale are estimated by shifting the jet momenta in the simulation up and down by one standard deviation of the jet energy corrections. Depending on the jet $p_{\mathrm {T}}$ and $\eta$, the resulting uncertainty in the simulated yields from the jet energy scale is typically 4%, except in the lowest regions in $M_{\text {T2}}(\ell \ell )$ close to the $m_{\text {W}}$ threshold where it can be as high as 20%. In addition, the energy scale of deposits from soft particles that are not clustered in jets are varied within their uncertainties, and the resulting uncertainty reaches 7%. The ${\text {b}}$tagging efficiency in the simulation is corrected using scale factors determined from the observed data [90], and uncertainties are propagated to all simulated events. These contribute an uncertainty of up to 7% in the predicted yields, depending on the $p_{\mathrm {T}}$, $\eta$ and origin of the ${\text {b}}$-tagged jet.

The effect of all the experimental uncertainties described above is evaluated for each of the simulated processes in all signal regions, and is considered correlated across the analysis bins and simulated processes.

**Fig. 4:**
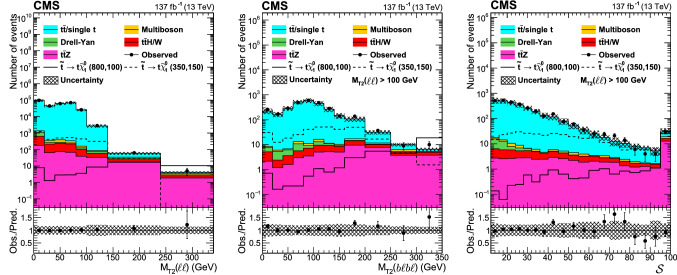
Distributions of $M_{\text {T2}}(\ell \ell )$ (left), $M_{\text {T2}}({\text {b}}\ell {\text {b}}\ell )$ (middle), and ${\mathcal {S}}$ (right) for all lepton flavors for the preselection defined in Table 2. Additionally, $M_{\text {T2}}(\ell \ell ) > 100\,\text {GeV}$ is required for the $M_{\text {T2}}({\text {b}}\ell {\text {b}}\ell )$ and ${\mathcal {S}}$ distributions. The last bin in each distribution includes the overflow events. The lower panel gives the ratio between the observation and the predicted SM backgrounds and the relative uncertainty in the SM background prediction is shown as a hatched band

**Fig. 5:**
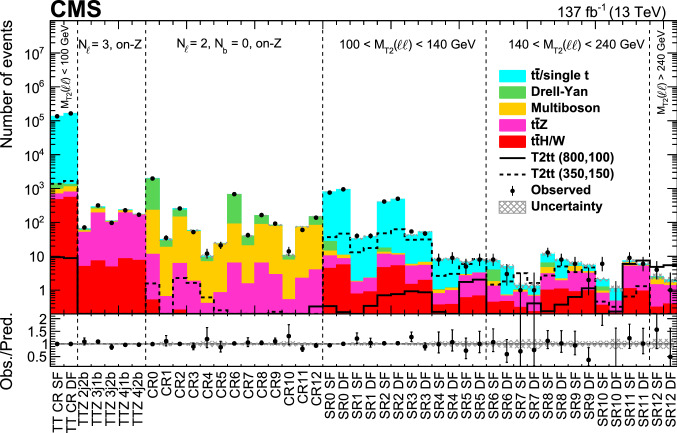
Predicted and observed yields in the signal and control regions as defined in Tables 3 and 4. The control regions TTCRSF and TTCRDF are defined by $M_{\text {T2}}(\ell \ell ) < 100\,\text {GeV}$ and are used to constrain the $\hbox {t}{\bar{\hbox {t}}}$ normalization. The $\hbox {t}{\bar{\hbox {t}}} {\text {Z}}$ control regions employ a 3 lepton requirement in different $N_\text {jets}$ and $N_{{\text {b}}}$ bins. The dilepton invariant mass and $N_{{\text {b}}}$ selections are inverted for CR0–CR12 in order to constrain the Drell–Yan and multiboson normalizations, using only the SF channel. The lower panel gives the ratio between the observation and the predicted SM backgrounds. The hatched band reflects the post-fit systematic uncertainties

**Fig. 6:**
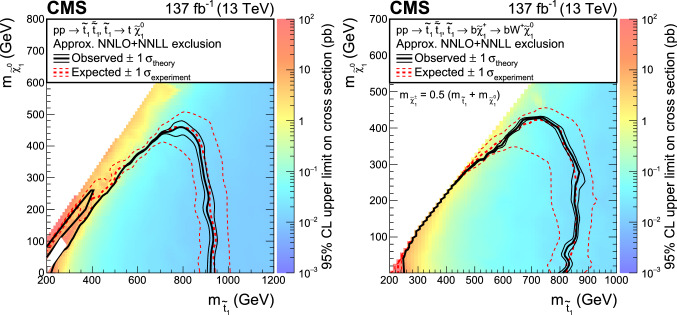
Expected and observed limits for the $\text {T2}{\text {t}}{\text {t}}$ model with $\tilde{{\text {t}}}_{1} \rightarrow {\text {t}}{\tilde{\chi }}^{0}_{1}$ decays (left) and for the $\text {T2}{{\text {b}}{\text {W}}}$ model with $\tilde{{\text {t}}}_{1} \rightarrow {\text {b}}{\tilde{\chi }}^{+}_{1} \rightarrow {\text {b}}\mathrm {W^+}{\tilde{\chi }}^{0}_{1}$ decays (right) in the $m_{\tilde{{\text {t}}}_{1}}$-$m_{{\tilde{\chi }}^{0}_{1}}$ mass plane. The color indicates the 95% $\text {CL}$ upper limit on the cross section at each point in the plane. The area below the thick black curve represents the observed exclusion region at 95% $\text {CL}$ assuming 100% branching fraction for the decays of the SUSY particles, while the dashed red lines indicate the expected limits at 95% $\text {CL}$ and the region containing 68% of the distribution of limits expected under the background-only hypothesis. The thin black lines show the effect of the theoretical uncertainties in the signal cross section. The small white area on the diagonal in the left figure corresponds to configurations where the mass difference between $\tilde{{\text {t}}}_{1}$ and ${\tilde{\chi }}^{0}_{1}$ is very close to the top quark mass. In this region the signal acceptance strongly depends on the ${\tilde{\chi }}^{0}_{1}$ mass and is therefore hard to model

The uncertainties in the normalizations of the single top and $\hbox {t}{\bar{\hbox {t}}}$, $\hbox {t}{\bar{\hbox {t}}} {\text {Z}}$, Drell–Yan, and multiboson backgrounds are discussed in Sect. 6. Finally, the uncertainty in the integrated luminosity is 2.3–2.5% [99–101].

Additional systematic uncertainties affect the modeling in simulation of the various processes, discussed in the following. All simulated samples are reweighted according to the distribution of the true number of interactions at each bunch crossing. The uncertainty in the total inelastic ${\text {p}}{\text {p}}$ cross section leads to uncertainties of 5% in the expected yields.

For the $\hbox {t}{\bar{\hbox {t}}}$ and $\hbox {t}{\bar{\hbox {t}}} {\text {Z}}$ backgrounds, we determine the event yield changes resulting from varying the renormalization scale ($\mu _\mathrm {R}$) and the factorization scale ($\mu _\mathrm {F}$) up and down by a factor of two, while keeping the overall normalization constant. The combinations of variations in opposite directions are disregarded. We assign as the uncertainty the envelope of the considered yield variations, treated as uncorrelated among the background processes. Uncertainties in the PDFs can have a further effect on the simulated $M_{\text {T2}}(\ell \ell )$ shape. We determine the change of acceptance in the signal regions using the PDF variations and assign the envelope of these variations—less than 4%—as a correlated uncertainty [102].

The contributions to the total uncertainty in the estimated backgrounds are summarized in Table 5, which provides the maximum uncertainties over all signal regions and the typical values, defined as the 90% quantile of the uncertainty values in all signal regions.

For the small contribution from $\hbox {t}{\bar{\hbox {t}}}$ production in association with a ${\text {W}}$or a Higgs boson, we take an uncertainty of 20% in the cross section based on the variations of the generator scales and the PDFs.

Most of the sources of systematic uncertainty in the background estimates affect the prediction of the signal as well, and these are evaluated separately for each mass configuration of the considered simplified models. We further estimate the effect of missing higher-order corrections for the signal acceptance by varying $\mu _\mathrm {R}$ and $\mu _\mathrm {F}$ [103–105] and find that those uncertainties are below 10%. The modeling of initial-state radiation (ISR) is relevant for the SUSY signal simulation in cases where the mass difference between the top squark and the LSP is small. The ISR reweighting is based on the number of ISR jets ($N_\mathrm {J}^{\text {ISR}}$) so as to make the predicted jet multiplicity distribution agree with that observed. The comparison is performed in a sample of events requiring two leptons and two ${\text {b}}$-tagged jets. The reweighting procedure is applied to SUSY MC events and factors vary between 0.92 and 0.51 for $N_\mathrm {J}^{\text {ISR}}$ between 1 and 6. We take one half of the deviation from unity as the systematic uncertainty in these reweighting factors, correlated across search regions. It is generally found to have a small effect, but can reach 30% for compressed mass configurations. An uncertainty from potential differences of the modeling of $p_{\mathrm {T}} ^{\text {miss}}$ in the fast simulation of the CMS detector is evaluated by comparing the reconstructed $p_{\mathrm {T}} ^{\text {miss}}$ with the $p_{\mathrm {T}} ^{\text {miss}}$ obtained using generator-level information. This uncertainty ranges up to 20% and only affects the SUSY signal samples. For these samples, the scale factors and uncertainties for the tagging efficiency of ${\text {b}}$jets and leptons are evaluated separately. Typical uncertainties in the scale factors are below 2% for ${\text {b}}$-tagged jets, and between 1 and 7% for leptons.

**Fig. 7:**
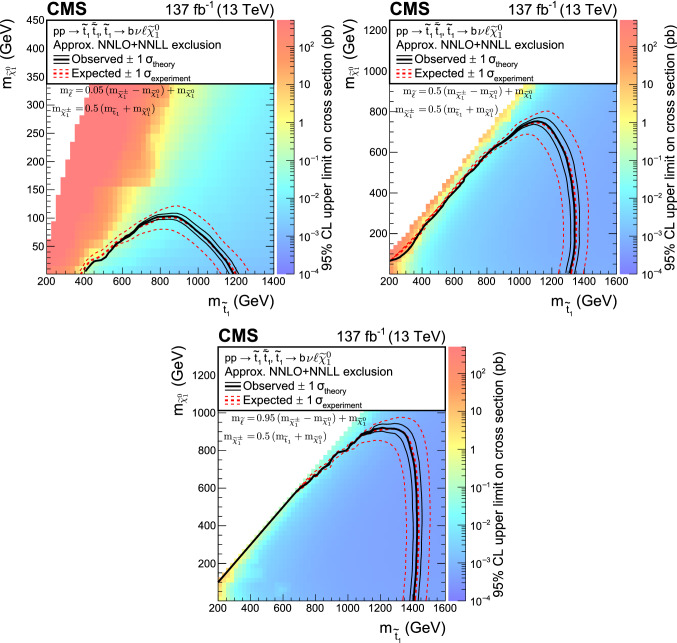
Expected and observed limits for the $\text {T8}{\text {b}}{\text {b}}\ell \ell \nu \nu$ model with $\tilde{{\text {t}}}_{1} \rightarrow {\text {b}}{\tilde{\chi }}^{+}_{1} \rightarrow {\text {b}}\nu {\widetilde{\ell }} \rightarrow {\text {b}}\nu \ell {\tilde{\chi }}^{0}_{1}$ decays in the $m_{\tilde{{\text {t}}}_{1}}$-$m_{{\tilde{\chi }}^{0}_{1}}$ mass plane for three different mass configurations defined by $m_{{\widetilde{\ell }}} = x \, (m_{{\tilde{\chi }}^{+}_{1}} - m_{{\tilde{\chi }}^{0}_{1}}) + m_{{\tilde{\chi }}^{0}_{1}}$ with $x=0.05$ (upper left), $x=0.50$ (upper right), and $x=0.95$ (lower). The description of curves is the same as in the caption of Fig. 6

## Results

Good agreement between the SM-predicted and observed $M_{\text {T2}}(\ell \ell )$, $M_{\text {T2}}({\text {b}}\ell {\text {b}}\ell )$, and ${\mathcal {S}}$ distributions is found, as shown in Fig. 4. No significant deviation from the SM prediction is observed in any of the signal regions as shown in Fig. 5. The observed excess events in SR10SF are found to be close to the signal region selection thresholds. To perform the statistical interpretations, a likelihood function is formed with Poisson probability functions for all data regions. The control and signal regions as depicted in Fig. 5 are included. The correlations of the uncertainties are taken into account as described in Sect. 7. A profile likelihood ratio in the asymptotic approximation [106] is used as the test statistic. Upper limits on the production cross section are calculated at 95% confidence level ($\text {CL}$) according to the asymptotic $\text {CL}_\text {s}$ criterion [107, 108].

The results shown in Fig. 5 are interpreted in the context of simplified SUSY models of top squark production followed by a decay to top quarks and neutralinos ($\text {T2}{\text {t}}{\text {t}}$), via an intermediate chargino ($\text {T2}{{\text {b}}{\text {W}}}$), and via an additional intermediate slepton ($\text {T8}{\text {b}}{\text {b}}\ell \ell \nu \nu$). These interpretations are presented on the $m_{\tilde{{\text {t}}}_{1}}$-$m_{{\tilde{\chi }}^{0}_{1}}$ plane in Figs. 6 and 7. The color on the *z* axis indicates the 95% $\text {CL}$ upper limit on the cross section at each point in the $m_{\tilde{{\text {t}}}_{1}}$-$m_{{\tilde{\chi }}^{0}_{1}}$ plane. The area below the thick black curve represents the observed exclusion region at 95% $\text {CL}$ assuming 100% branching fraction for the decays of the SUSY particles. The thick dashed red lines indicate the expected limit at 95% $\text {CL}$, while the region containing 68% of the distribution of limits expected under the background-only hypothesis is bounded by thin dashed red lines. The thin black lines show the effect of the theoretical uncertainties in the signal cross section. In the $\text {T2}{\text {t}}{\text {t}}$ model we exclude mass configurations with $m_{{\tilde{\chi }}^{0}_{1}}$ up to 450$\,\text {GeV}$ and $m_{\tilde{{\text {t}}}_{1}}$ up to 925$\,\text {GeV}$, assuming that the top quarks are unpolarized, thus improving by approximately 125$\,\text {GeV}$ in $m_{\tilde{{\text {t}}}_{1}}$ the results presented on a partial data set in Ref. [38]. The observed upper limit on the top squark cross section improved by approximately 50% for most mass configurations. The result for the $\text {T2}{{\text {b}}{\text {W}}}$ model is shown in Fig. 6 (right) and the results for $\text {T8}{\text {b}}{\text {b}}\ell \ell \nu \nu$ models are shown in Fig. 7. We exclude mass configurations with $m_{{\tilde{\chi }}^{0}_{1}}$ up to 420$\,\text {GeV}$ and $m_{\tilde{{\text {t}}}_{1}}$ up to 850$\,\text {GeV}$ in the $\text {T2}{{\text {b}}{\text {W}}}$ model, extending the exclusion limits set in Ref. [38] by approximately 100$\,\text {GeV}$ in $m_{\tilde{{\text {t}}}_{1}}$. The sensitivity in the $\text {T8}{\text {b}}{\text {b}}\ell \ell \nu \nu$ model strongly depends on the intermediate slepton mass and is largest when $x = 0.95$ in $m_{{\widetilde{\ell }}} = x \, (m_{{\tilde{\chi }}^{+}_{1}} - m_{{\tilde{\chi }}^{0}_{1}}) + m_{{\tilde{\chi }}^{0}_{1}}$. In this case, excluded masses reach up to 900$\,\text {GeV}$ for $m_{{\tilde{\chi }}^{0}_{1}}$ and 1.4$\,\text {TeV}$ for $m_{\tilde{{\text {t}}}_{1}}$. These upper limits decrease to 750$\,\text {GeV}$ for $m_{{\tilde{\chi }}^{0}_{1}}$ and 1.3$\,\text {TeV}$ for $m_{\tilde{{\text {t}}}_{1}}$ when $x=0.5$ and to 100$\,\text {GeV}$ for $m_{{\tilde{\chi }}^{0}_{1}}$ and 1.2$\,\text {TeV}$ for $m_{\tilde{{\text {t}}}_{1}}$ when $x=0.05$. In this model, the improvement upon previous results from Ref. [38] is approximately 100$\,\text {GeV}$ in $m_{\tilde{{\text {t}}}_{1}}$, and up to 100$\,\text {GeV}$ in $m_{{\tilde{\chi }}^{0}_{1}}$.

## Summary

A search for top squark pair production in final states with two opposite-charge leptons, ${\text {b}}$jets, and significant missing transverse momentum ($p_{\mathrm {T}} ^{\text {miss}}$) is presented. The data set of proton-proton collisions corresponds to an integrated luminosity of 137$\,{\text {fb}}^{-1}$ and was collected with the CMS detector at a center-of-mass energy of 13$\,\text {TeV}$. Transverse mass variables and the significance of $p_{\mathrm {T}} ^{\text {miss}}$ are used to efficiently suppress backgrounds from standard model processes. No evidence for a deviation from the expected background is observed. The results are interpreted in several simplified models for supersymmetric top squark pair production and decay.

In the $\text {T2}{\text {t}}{\text {t}}$ model with $\tilde{{\text {t}}}_{1} \rightarrow {\text {t}}{\tilde{\chi }}^{0}_{1}$ decays, $\tilde{{\text {t}}}_{1}$ masses up to 925$\,\text {GeV}$ and ${\tilde{\chi }}^{0}_{1}$ masses up to 450$\,\text {GeV}$ are excluded. In the $\text {T2}{{\text {b}}{\text {W}}}$ model with $\tilde{{\text {t}}}_{1} \rightarrow {\text {b}}{\tilde{\chi }}^{+}_{1} \rightarrow {\text {b}}\mathrm {W^+}{\tilde{\chi }}^{0}_{1}$ decays, $\tilde{{\text {t}}}_{1}$ masses up to 850$\,\text {GeV}$ and ${\tilde{\chi }}^{0}_{1}$ masses up to 420$\,\text {GeV}$ are excluded, assuming the chargino mass to be the mean of the $\tilde{{\text {t}}}_{1}$ and ${\tilde{\chi }}^{0}_{1}$ masses. In the $\text {T8}{\text {b}}{\text {b}}\ell \ell \nu \nu$ model with decays $\tilde{{\text {t}}}_{1} \rightarrow {\text {b}}{\tilde{\chi }}^{+}_{1} \rightarrow {\text {b}}\nu {\widetilde{\ell }} \rightarrow {\text {b}}\nu \ell {\tilde{\chi }}^{0}_{1}$, therefore 100% branching fraction to dilepton final states, the sensitivity depends on the intermediate particle masses. With the chargino mass again taken as the mean of the $\tilde{{\text {t}}}_{1}$ and ${\tilde{\chi }}^{0}_{1}$ masses, the strongest exclusion is obtained if the slepton mass is close to the chargino mass. In this case, excluded masses reach up to 1.4$\,\text {TeV}$ for $\tilde{{\text {t}}}_{1}$ and 900$\,\text {GeV}$ for ${\tilde{\chi }}^{0}_{1}$. When the slepton mass is taken as the mean of the chargino and neutralino masses, these numbers decrease to 1.3$\,\text {TeV}$ for $\tilde{{\text {t}}}_{1}$ and 750$\,\text {GeV}$ for ${\tilde{\chi }}^{0}_{1}$. A further reduction to 1.2$\,\text {TeV}$ for $\tilde{{\text {t}}}_{1}$ and to 100$\,\text {GeV}$ for ${\tilde{\chi }}^{0}_{1}$ is observed when the slepton mass is close to the neutralino mass.

## Data Availability

This manuscript has no associated data or the data will not be deposited. [Authors’ comment: Release and preservation of data used by the CMS Collaboration as the basis for publications is guided by the CMS policy as written in its document “CMS data preservation, re-use and open access policy” (https://cms-docdb.cern.ch/cgi-bin/PublicDocDB/RetrieveFile?docid=6032&filename=CMSDataPolicyV1.2.pdf&version=2).].
